# Glutathione Biology in Neurodegenerative and Metabolic Diseases: Molecular Mechanisms, Pathophysiological Roles, and Therapeutic Perspectives

**DOI:** 10.3390/ijms27167507

**Published:** 2026-08-21

**Authors:** Grażyna Gromadzka, Magdalena Kąkol, Magdalena Klimkiewicz, Maria Bendykowska

**Affiliations:** 1 Department of Biomedical Sciences, Faculty of Medicine, Collegium Medicum, Cardinal Stefan Wyszynski University in Warsaw, Wóycickiego Street 1/3, 01-938 Warsaw, Poland; 2 Student Scientific Society “Immunis”, Faculty of Medicine, Collegium Medicum, Cardinal Stefan Wyszynski University in Warsaw, Wóycickiego Street 1/3, 01-938 Warsaw, Poland; 3 Military Institute of Medicine, National Research Institute, Szaserów Street 128, 04-141 Warsaw, Poland

**Keywords:** glutathione, redox homeostasis, oxidative stress, Nrf2, mitochondrial glutathione, S-glutathionylation, neurodegenerative diseases, metabolic diseases, antioxidant therapy

## Abstract

Glutathione is an abundant intracellular low-molecular-weight thiol that contributes importantly to cellular redox homeostasis. Besides its well-established role in the antioxidant defense of the cell, glutathione regulates mitochondrial function, metabolism of toxicants, protein thiol oxidation/reduction, redox signaling, and immunity. Disturbances in glutathione metabolism have been shown to play a role in various diseases; however, it has become clear that changes in glutathione metabolism are a part of a complex, multifactorial process. In this review, we summarize current knowledge of the molecular mechanisms governing glutathione synthesis, recycling, compartmentalization, and biological functions, with particular emphasis on redox signaling, the nuclear factor erythroid 2-related factor 2/Kelch-like ECH-associated protein 1 (Nrf2/Keap1) pathway, and reversible protein S-glutathionylation. We further examine how disturbances in glutathione homeostasis interact with mitochondrial dysfunction, chronic inflammation, metabolic stress, and impaired cellular signaling in Parkinson’s disease, Alzheimer’s disease, Huntington’s disease, multiple sclerosis, Wilson’s disease, type 2 diabetes, and nonalcoholic fatty liver disease. We also evaluate current translational interventions targeting restoration of glutathione balance through glutathione supplementation, precursor supplementation, pharmacological modulation of endogenous antioxidant mechanisms, dietary interventions, and changes in lifestyle. Despite the fact that many interventions have been promising at the mechanistic and experimental level, there are still insufficient clinical data because of the problems associated with glutathione availability, tissue specificity, disease variability, and a lack of sufficiently powered clinical trials. The conclusion of this review is that glutathione should not be viewed as a universal therapeutic target; instead, glutathione should be perceived as an important factor contributing to cellular resilience and able to help other disease-specific interventions. Future progress in glutathione-based interventions will likely depend on integrating redox biomarkers, patient stratification, and precision medicine strategies to identify individuals most likely to benefit from targeted modulation of glutathione homeostasis.

## 1. Introduction

Glutathione is a tripeptide composed of glutamate, cysteine, and glycine and is a major intracellular non-protein thiol involved in the regulation of cellular redox homeostasis. Besides its role in the direct elimination of reactive oxygen species (ROS) and reactive nitrogen species (RNS), glutathione is involved in detoxification, protein thiol maintenance, mitochondrial activity, regulation of redox-dependent signaling pathways, and immune function [1,2]. Consequently, maintenance of intracellular glutathione homeostasis is essential for preserving normal cellular physiology.

The intracellular redox environment is commonly assessed using the ratio of reduced glutathione (GSH) to oxidized glutathione (GSSG), which reflects the balance between antioxidant defenses and oxidative burden [3,4]. Disruptions in this balance contribute to protein, lipid, and DNA oxidation and may affect a number of signaling pathways involved in cellular survival, inflammation, metabolism, and apoptosis [1,4]. However, oxidative stress should be regarded as an important component of complex cellular pathology rather than an isolated pathogenic mechanism.

Particularly vulnerable to disturbances in redox homeostasis are tissues with high metabolic demands [5,6]. In the case of the central nervous system (CNS), the excessive formation of ROS leads to mitochondrial impairment and triggers the activation of microglia and astrocytes, which in turn promotes the production of cytokines like interleukin-1β (IL-1β), tumor necrosis factor-α (TNF-α), and interleukin-6 (IL-6) [6,7,8,9,10]. These processes are increasingly recognized as interconnected components of neurodegeneration rather than independent pathological events [7,8,9].

Experimental and clinical evidence suggests that alterations in glutathione metabolism may be responsible for diverse chronic diseases [11,12,13]. For example, in Parkinson’s disease (PD), a reduction in glutathione concentration has been observed in substantia nigra at an early stage in the development of the disease, which may result in greater susceptibility of neurons to oxidative stress [10,14,15]. In Alzheimer’s disease (AD), impaired glutathione homeostasis has been associated with increased oxidative damage and mitochondrial dysfunction, although its precise causal contribution remains incompletely understood [16,17]. Likewise, patients with type 2 diabetes (T2D) frequently exhibit decreased glutathione levels together with chronic redox imbalance, inflammation, and metabolic dysregulation [18,19,20]. Nevertheless, in each of these disorders, glutathione dysregulation represents only one element of a multifactorial pathogenic network involving genetic susceptibility, mitochondrial dysfunction, altered proteostasis, immune activation, metabolic abnormalities, and environmental influences.

In the last decade, there has been increased interest in glutathione not only for its role as a cellular antioxidant but also because of its function as a key player in redox regulation. Through S-glutathionylation of proteins in a reversible manner, regulation of transcription factors like nuclear factor erythroid 2-related factor 2 (Nrf2), and regulation of mitochondrial redox homeostasis, glutathione regulates many adaptive cellular processes that go beyond reactive oxygen species scavenging [10,15,21,22,23]. Emerging evidence further highlights the importance of glutathione compartmentalization, particularly the mitochondrial glutathione pool. The mGSH pool is independently regulated and appears to be particularly important for maintaining mitochondrial bioenergetics and preventing oxidative injury despite mitochondria lacking the capacity for de novo GSH synthesis. Disruption of mitochondrial glutathione homeostasis has therefore emerged as a potentially important contributor to several neurodegenerative and metabolic disorders [24].

Glutathione has been widely researched as a potential therapeutic target, although its effective application has proven difficult to achieve. The oral delivery of glutathione is hampered by poor bioavailability, whereas approaches involving precursors, diet, and drug stimulation of antioxidant mechanisms are not consistent in their efficacy due to dependence on the specific disease setting [25,26,27,28]. Thus, the restoration of glutathione homeostasis may currently be considered as an additional therapy, not as a disease-modifying intervention with clinically proven efficacy.

Therefore, the aim of this review is to summarize current knowledge on the biology of glutathione as well as to critically evaluate and integrate the available evidence regarding its role in neurodegenerative and metabolic diseases. We discuss the molecular regulation of glutathione metabolism, specifics of glutathione functions at the tissue level, participation of glutathione in the pathogenesis of neurodegenerative and metabolic diseases as part of multifactorial pathophysiology, and potential and existing challenges associated with the development of glutathione-targeted therapies. Particular emphasis is placed on identifying knowledge gaps that may guide future translational research.

## 2. Materials and Methods

This review was prepared using a structured narrative approach with elements of systematic literature selection to provide a comprehensive and critical overview of current knowledge regarding glutathione biology, its role in neurodegenerative and metabolic diseases, and its translational potential. The review was developed in accordance with the recommendations of the Scale for the Assessment of Narrative Review Articles (SANRA), which guided its overall structure, critical appraisal of the literature, and presentation of evidence. Rather than attempting a quantitative synthesis of the available evidence, the objective was to integrate mechanistic, experimental, and clinical findings while highlighting areas of consensus, controversy, and remaining knowledge gaps.

### 2.1. Literature Search Strategy

A comprehensive literature search was performed using the PubMed/MEDLINE, Scopus, and Web of Science databases for studies published up to March 2026. Search terms comprised combinations of Medical Subject Headings (MeSH) and free-text keywords, including “glutathione”, “GSH”, “oxidative stress”, “redox homeostasis”, “Nrf2”, “Keap1”, “S-glutathionylation”, “mitochondrial glutathione”, “neurodegeneration”, “Parkinson’s disease”, “Alzheimer’s disease”, “Huntington’s disease”, “Wilson’s disease”, “multiple sclerosis”, “type 2 diabetes”, “nonalcoholic fatty liver disease”, “antioxidant therapy”, and “GlyNAC”. Boolean operators (AND, OR) were applied to optimize search specificity and sensitivity.

### 2.2. Eligibility Criteria

The review included:

- Original experimental studies;
- Clinical studies;
- Systematic reviews and meta-analyses;
- High-quality narrative reviews relevant to glutathione biology.

Priority was given to studies published within the last 5–10 years, while seminal earlier publications were retained when essential for understanding fundamental biochemical mechanisms. Studies were selected based on their mechanistic relevance, methodological quality, and contribution to current understanding of glutathione metabolism or therapeutic modulation.

Conference abstracts without full-text publications, non-English articles, and studies lacking mechanistic or translational relevance were excluded.

### 2.3. Data Extraction and Critical Synthesis

Selected publications were evaluated, with particular attention paid to the following:

- Molecular mechanisms regulating glutathione synthesis, recycling, transport, and compartmentalization;
- Redox-sensitive signaling pathways, including Nrf2/Kelch-like ECH-associated protein 1 (Nrf2/Keap1) and nuclear factor kappa-B (NF-κB);
- Tissue- and cell-specific regulation of glutathione metabolism;
- Evidence linking glutathione dysregulation with disease pathophysiology;
- Preclinical and clinical studies evaluating glutathione-targeted interventions.

Unlike the descriptive summary of individual research studies, the evidence synthesis involved the integration of information based on the strength of the evidence. The synthesis put special emphasis on the difference between results that were clearly substantiated by clinical evidence and those obtained mainly from experimental research or translational research at an earlier stage.

## 3. Glutathione: Metabolism, Redox Regulation, and Tissue-Specific Functions

### 3.1. Glutathione Biosynthesis, Redox Cycling, and Cellular Regulation

Glutathione is synthesized exclusively within cells through a tightly regulated two-step ATP-dependent enzymatic pathway [29,30]. The rate-limiting step is catalyzed by glutamate–cysteine ligase (GCL). It condenses glutamate and cysteine to form γ-glutamylcysteine [31,32,33,34]. GCL consists of catalytic (GCLC) and modifier (GCLM) subunits whose coordinated regulation determines overall enzyme activity [31,35,36,37]. The second reaction is catalyzed by glutathione synthetase (GS), which incorporates glycine to generate GSH [38,39,40]. Defects in either enzyme impair intracellular glutathione availability and increase cellular susceptibility to oxidative injury [31,41,42].

Following detoxification of ROS or participation in glutathione S-transferase-mediated conjugation reactions, GSH is oxidized to GSSG. Regeneration of GSH is catalyzed by the glutathione reductase (GR) in a nicotinamide adenine dinucleotide phosphate (NADPH)-dependent reaction that maintains the high intracellular GSH/GSSG ratio characteristic of healthy cells [43,44]. Consequently, adequate NADPH production through the pentose phosphate pathway is essential for preserving glutathione homeostasis during oxidative stress.

A major regulatory mechanism controlling glutathione metabolism is the Keap1/Nrf2/antioxidant response element (ARE) signaling pathway [45,46,47,48,49]. Under basal conditions, Nrf2 undergoes continuous ubiquitination and proteasomal degradation through interaction with Keap1. Oxidative or electrophilic stress modifies critical cysteine residues within Keap1, allowing Nrf2 stabilization and nuclear translocation, where it activates ARE-containing genes, including *GCLC*, *GCLM*, glutathione reductase gene (*GSR*), glutathione peroxidase gene (*GPX*), and genes encoding several glutathione S-transferases [45,46,48,49,50,51]. Importantly, activation of Nrf2 coordinates a broad cytoprotective program extending beyond glutathione synthesis, including xenobiotic detoxification, NADPH generation, and mitochondrial protection [52].

Besides its antioxidant function, glutathione also regulates intracellular signaling through reversible protein S-glutathionylation [53,54,55,56,57].

S-Glutathionylation serves as a reversible protective shield for highly reactive cysteines against permanent oxidation and simultaneously regulates enzyme function, protein stability, mitochondrial metabolism, inflammation, and cell death. S-Glutathionylation should not be perceived only as an effect of oxidative stress but rather as an important physiological redox signaling mechanism [53].

Another increasingly recognized aspect of glutathione biology is intracellular compartmentalization. Although synthesized exclusively in the cytosol, glutathione is actively transported into mitochondria. There it forms a distinct mitochondrial glutathione (mGSH) pool that is regulated independently of the cytosolic compartment [58,59,60,61].

Because mitochondria cannot synthesize glutathione de novo, maintenance of mGSH depends entirely on specialized carrier systems located in the inner mitochondrial membrane. Depletion of mGSH has been associated with increased mitochondrial ROS generation, impaired oxidative phosphorylation, altered calcium homeostasis, and enhanced susceptibility to apoptosis in numerous experimental models. An increasing body of evidence suggests that selective disruption of mitochondrial glutathione balance may represent an early pathological event in several neurodegenerative and metabolic disorders, which makes mitochondria an emerging pharmacological target [62,63,64].

In summary, all these findings demonstrate that compartmentation of glutathione adds another level of redox control to the total GSH levels in cells.

**Figure 1:**
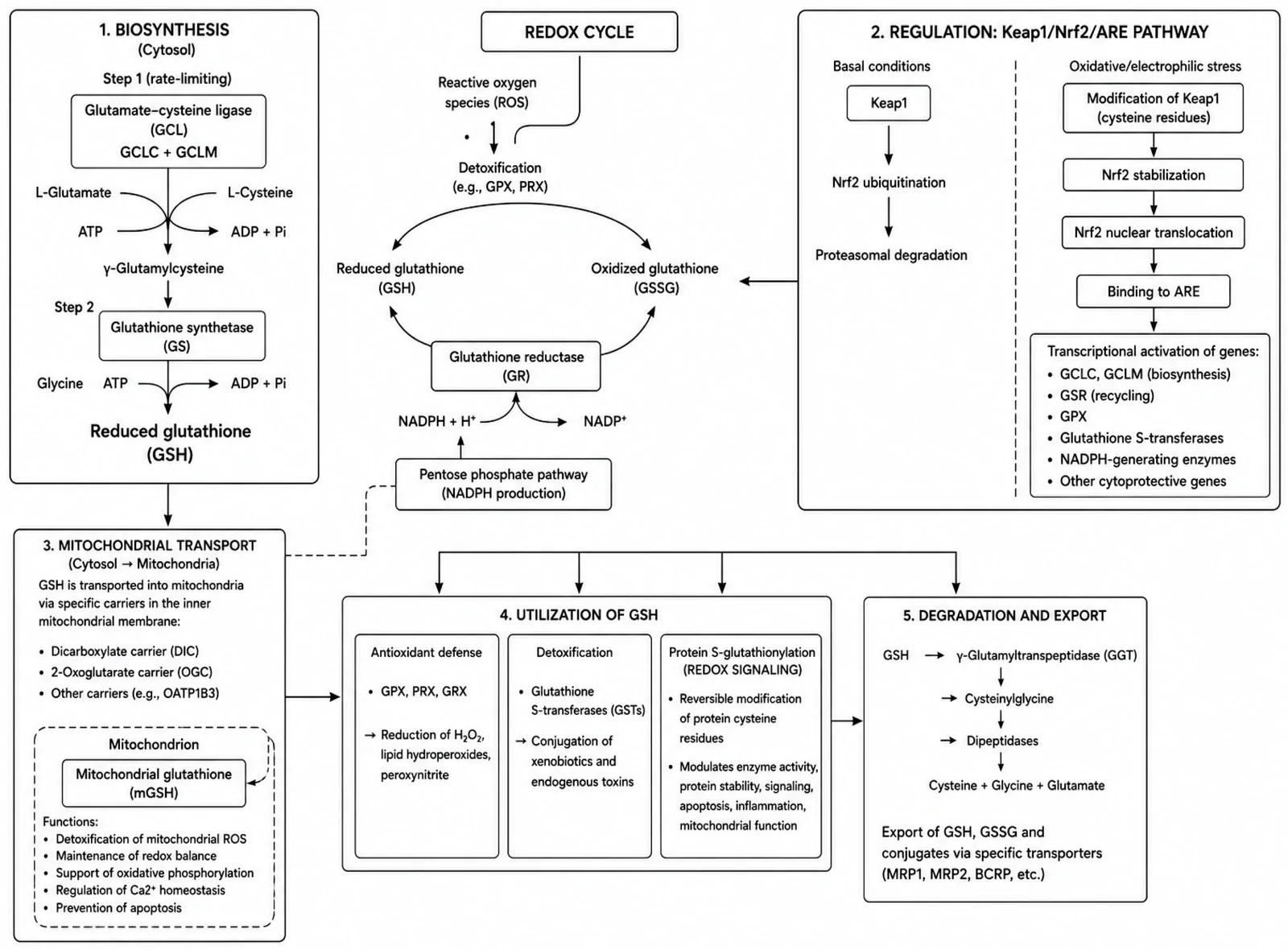
Molecular overview of glutathione metabolism and redox homeostasis. Abbreviations: ADP, adenosine diphosphate; ARE, antioxidant response element; ATP, adenosine triphosphate; BCRP, breast cancer resistance protein; DIC, dicarboxylate carrier; GCL, glutamate–cysteine ligase; GCLC, catalytic subunit of glutamate–cysteine ligase; GCLM, modifier subunit of glutamate–cysteine ligase; GPX, glutathione peroxidase; GR, glutathione reductase; GRX, glutaredoxin; GSH, reduced glutathione; GSR, glutathione reductase; GSSG, oxidized glutathione (glutathione disulfide); GST, glutathione S-transferase; Keap1, Kelch-like ECH-associated protein 1; mGSH, mitochondrial glutathione; MRP, multidrug resistance-associated protein; NADP^+^, oxidized nicotinamide adenine dinucleotide phosphate; NADPH, reduced nicotinamide adenine dinucleotide phosphate; Nrf2, nuclear factor erythroid 2–related factor 2; OATP1B3, organic anion transporting polypeptide 1B3; OGC, 2-oxoglutarate carrier; Pi, inorganic phosphate; PRX, peroxiredoxin; ROS, reactive oxygen species. In all figures, solid arrows indicate established mechanisms, whereas dashed arrows represent indirect interactions, feedback loops, or mechanisms that remain incompletely understood.

Table 1 summarizes the major enzymes involved in glutathione biosynthesis, regeneration, utilization, and degradation. Figure 1 integrates the key molecular pathways underlying GSH metabolism, including biosynthesis, redox cycling, intracellular compartmentalization, Keap1/Nrf2/ARE signaling, and the biological processes that collectively maintain cellular redox homeostasis.

### 3.2. Tissue Distribution and Intracellular Compartmentalization of Glutathione

Intracellular glutathione concentrations vary considerably among tissues and cell types. This variation reflects differences in metabolic activity, mitochondrial content, exposure to oxidants, and antioxidant requirements [65,66,67]. Although GSH is present in virtually all mammalian cells, its abundance and functional importance are highly tissue specific. High glutathione concentrations are found in organs with high oxidative metabolic activity. These include the liver, kidneys, lungs, and brain, because they are exposed to sustained oxidative stress and electrophilic compounds [68,69,70].

The liver contains the highest GSH concentration in the body and serves as the principal organ responsible for glutathione synthesis and systemic distribution [39,71]. Glutathione in the liver not only helps maintain redox balance but also influences detoxification reactions catalysed by the enzyme glutathione S-transferase. Moreover, glutathione is secreted by hepatocytes into blood plasma and bile [72,73].

High concentrations of GSH in the epithelial lining fluid protect pulmonary epithelial cells from oxidative injury and help maintain epithelial barrier integrity. Glutathione alterations within the lungs have been associated with an increased risk for developing chronic respiratory diseases, where oxidative stress interacts with inflammation and environmental factors as opposed to being an independent pathogenic process [74,75].

The kidneys also contain abundant glutathione because of their high metabolic activity and central role in filtration, transport, and xenobiotic metabolism. Renal GSH participates in detoxification reactions and protects tubular epithelial cells from oxidative damage generated during active solute transport and exposure to endogenous and exogenous toxins [76,77].

Within the CNS, glutathione metabolism displays marked cell-type specificity [78]. Astrocytes synthesize and store substantially larger amounts of GSH than neurons. They provide cysteine precursors that support neuronal glutathione synthesis through metabolic coupling [78,79]. Consequently, neurons rely heavily on astrocyte-mediated maintenance of antioxidant capacity, making disruption of neuron-glia metabolic interactions an important contributor to disturbed redox homeostasis in neurodegenerative disorders [78]. Oligodendrocytes and microglia also exhibit distinct patterns of glutathione dynamics that influence their responses to inflammation, myelin maintenance, and oxidative injury [78,80]. These cell-specific differences help explain why individual neurological diseases affect distinct cellular populations despite sharing common features of redox imbalance.

Glutathione distribution is also highly compartmentalized within individual cells. Although synthesized exclusively in the cytosol, GSH is transported into mitochondria, the nucleus, endoplasmic reticulum, and peroxisomes [58,62,81,82].

Among these compartments, mGSH is of particular importance because mitochondria generate large amounts of ROS while lacking the capacity for de novo GSH synthesis. As discussed in Section 3.1, maintenance of mGSH depends on specialized transport systems located in the inner mitochondrial membrane [58,59,60,61].

Adequate mGSH concentrations are essential for preserving oxidative phosphorylation, regulating mitochondrial permeability transition, maintaining calcium homeostasis, and limiting mitochondrial DNA damage [62,63,83,84]. Consequently, tissue-specific differences in mitochondrial glutathione availability may substantially influence susceptibility to oxidative injury. Cells with high oxidative metabolism—such as neurons, hepatocytes, cardiomyocytes, and skeletal muscle fibers—rely heavily on adequate mGSH to preserve mitochondrial function and bioenergetic homeostasis. Conversely, depletion of mitochondrial glutathione may render these tissues particularly vulnerable to oxidative stress and contribute to disease-specific pathological mechanisms [83].

Glutathione distribution between tissues and subcellular organelles is essential in determining their sensitivity to oxidative damage. Nevertheless, glutathione levels in tissues cannot be used as an indicator of disease vulnerability. Disease vulnerability is associated with a combination of multiple factors including antioxidant status, metabolic rate, mitochondrial status, inflammatory response, regeneration ability, and molecular processes associated with diseases. Thus, even though the vulnerability of some tissues is connected to their lower glutathione levels, glutathione depletion should be regarded as just one of the factors in pathogenesis.

For clarity, Table 2 summarizes tissue-specific glutathione concentrations and their principal physiological functions.

## 4. Glutathione in Neurodegenerative Diseases

### 4.1. Parkinson’s Disease

PD is a progressive neurodegenerative disorder characterized by the preferential loss of dopaminergic neurons within the substantia nigra pars compacta (SNpc) and the accumulation of misfolded α-synuclein in Lewy bodies [85,86,87]. Although oxidative stress is widely recognized as an important contributor to PD pathophysiology, the disease is multifactorial. It involves complex interactions among genetic susceptibility, mitochondrial dysfunction, impaired proteostasis, neuroinflammation, altered dopamine metabolism, and age-related decline in cellular homeostasis [88,89,90].

As discussed in Section 3, glutathione is essential for maintaining neuronal redox homeostasis. Among neurodegenerative disorders, PD is unique because marked depletion of glutathione has been consistently detected in the substantia nigra during the early stages of disease progression, preceding extensive neuronal loss [14,91,92,93,94]. This selective reduction in glutathione suggests that impaired redox homeostasis may increase the vulnerability of dopaminergic neurons to subsequent pathogenic insults. However, whether glutathione depletion represents a primary pathogenic event or an early consequence of mitochondrial dysfunction remains incompletely resolved.

The selective susceptibility of dopaminergic neurons is closely related to their distinctive metabolic characteristics. Dopamine metabolism itself generates reactive oxygen species and reactive dopamine quinones, while these neurons simultaneously rely heavily on mitochondrial oxidative phosphorylation to meet their high energy demands. Consequently, even relatively modest impairment of antioxidant defences may result in excessive oxidative burden. Mitochondrial complex I dysfunction, consistently observed in postmortem studies and experimental models of PD, further enhances ROS production and compromises ATP generation, thereby creating conditions that favour progressive neuronal injury [92,95,96,97]. Thus, oxidative stress develops within a cellular environment that is already characterized by limited antioxidant reserve and high energetic demand.

Another hallmark of PD is the bidirectional relationship between disturbed redox homeostasis and α-synuclein pathology. Experimental studies have demonstrated that ROS and lipid peroxidation products, including 4-hydroxynonenal (4-HNE), promote post-translational modifications of α-synuclein, facilitating its oligomerization and aggregation [89,98,99]. In turn, aggregated α-synuclein further disrupts mitochondrial function, impairs proteostasis, and enhances oxidative stress, creating a self-perpetuating pathogenic cycle that contributes to dopaminergic neurodegeneration [100,101].

Besides reducing antioxidant buffering capacity, glutathione dysregulation may also influence redox-sensitive signaling through reversible protein S-glutathionylation, thereby affecting the function of proteins involved in mitochondrial metabolism and cellular stress responses [10,93,102]. In parallel, chronic microglial activation and sustained production of pro-inflammatory mediators amplify oxidative injury, further increasing neuronal vulnerability [103].

**Figure 2:**
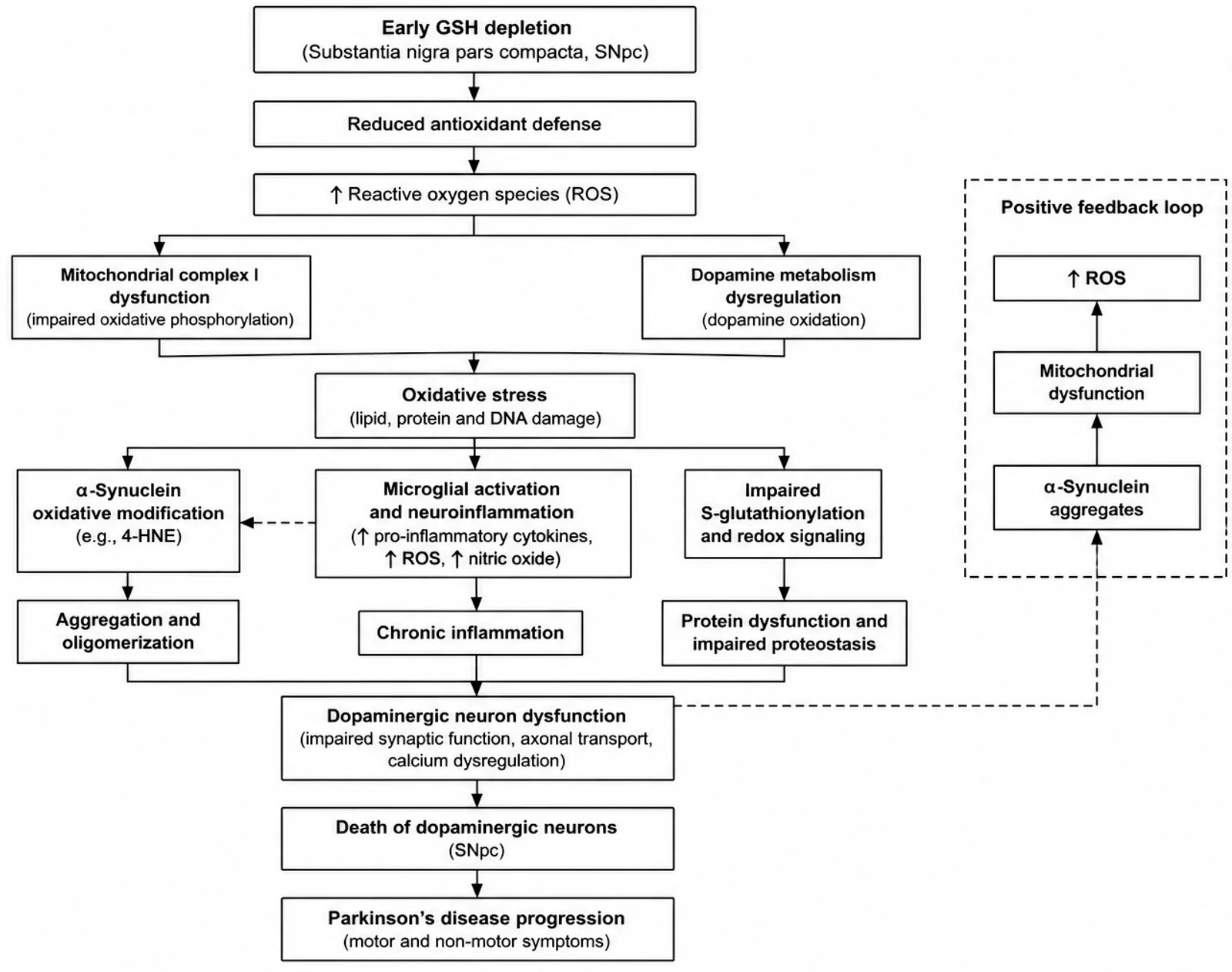
Glutathione dysregulation and oxidative stress in Parkinson’s disease. Abbreviations: 4-HNE, 4-hydroxynonenal; GSH, reduced glutathione; ROS, reactive oxygen species; SNpc, substantia nigra pars compacta.

These interrelated processes are illustrated in Figure 2. Based on the above information, it may be suggested that the disturbance of glutathione regulation is an integral modulator of PD pathophysiology rather than an independent process.

Experimental studies indicate that restoration of glutathione homeostasis through glutathione precursors such as N-acetylcysteine (NAC) or pharmacological activation of the Nrf2 pathway may reduce redox imbalance and improve mitochondrial function in cellular and animal models of PD [93,100,102]. However, clinical evidence remains limited, and current data are insufficient to demonstrate disease-modifying efficacy in patients. Even though some preliminary results of clinical investigations have indicated a positive effect on certain parameters, further research is required before drawing definite conclusions.

### 4.2. Alzheimer’s Disease

AD is the most common cause of dementia. It is characterized by progressive cognitive decline associated with extracellular deposition of amyloid-β (Aβ) plaques, intracellular neurofibrillary tangles composed of hyperphosphorylated tau protein, synaptic dysfunction, and neuronal loss [104,105,106]. Current evidence indicates that it develops through complex interactions among protein aggregation, mitochondrial dysfunction, impaired proteostasis, neuroinflammation, vascular alterations, and age-related metabolic changes, with oxidative stress representing an important but non-exclusive component of this multifactorial process [107,108,109,110].

As discussed in Section 3, glutathione is a major determinant of neuronal redox homeostasis. Several experimental and clinical studies have reported reduced GSH levels in the brains of patients with AD, particularly in regions vulnerable to neurodegeneration such as the hippocampus and cerebral cortex [111,112,113]. Magnetic resonance spectroscopy studies further suggest that alterations in glutathione metabolism may occur during the early stages of cognitive decline, although reported findings remain partially inconsistent because of methodological differences and disease heterogeneity [111,113,114].

A defining feature of AD is the progressive accumulation of Aβ and hyperphosphorylated tau, which together drive synaptic dysfunction and neuronal degeneration. Oxidative stress has been shown to facilitate amyloidogenic processing of amyloid precursor protein (APP), thereby increasing Aβ production [115,116,117]. In parallel, disturbed redox homeostasis facilitates tau hyperphosphorylation and aggregation, contributing to cytoskeletal disruption, impaired axonal transport, and progressive loss of neuronal connectivity [115,116,117,118]. Rather than acting independently, these pathological processes reinforce one another, creating a self-amplifying network that ultimately leads to synaptic failure and cognitive decline.

Within this context, glutathione appears to function primarily as a modulator of neuronal resilience rather than a direct determinant of disease initiation. Reduced GSH availability may diminish antioxidant buffering capacity, alter redox-sensitive signaling pathways, and disturb reversible protein S-glutathionylation, thereby affecting enzymes involved in energy metabolism, cytoskeletal organization, and synaptic transmission [119,120,121]. Consequently, glutathione deficiency may facilitate the progression of Aβ- and tau-mediated pathology rather than initiate these pathological events. Moreover, the chronic activation of microglia and astrocytes leads to increased production of pro-inflammatory cytokines and oxidative/nitrosative stress molecules, resulting in increased neurodegeneration through mechanisms that have close interplay with mitochondrial abnormalities and protein aggregation [103,122].

Experimental studies have also demonstrated impaired activation of the Nrf2 pathway in AD, potentially reducing the expression of antioxidant enzymes and limiting endogenous glutathione synthesis [123,124,125]. Pharmacological activation of Nrf2 restores antioxidant capacity and improves mitochondrial function in numerous experimental models [126,127,128].

Accordingly, several strategies aimed at supporting glutathione homeostasis—including glutathione supplementation, NAC administration, dietary antioxidants, and pharmacological activation of endogenous antioxidant pathways—have been investigated [10,129,130]. Although many of these approaches improve biochemical markers of oxidative stress in experimental studies, their translation into meaningful clinical benefit has been limited by the complexity of AD pathophysiology, poor brain bioavailability of glutathione, and considerable inter-individual variability [80,131]. At present, glutathione-targeted interventions should therefore be regarded as adjunctive strategies intended to enhance neuronal resilience rather than disease-modifying therapies.

**Figure 3:**
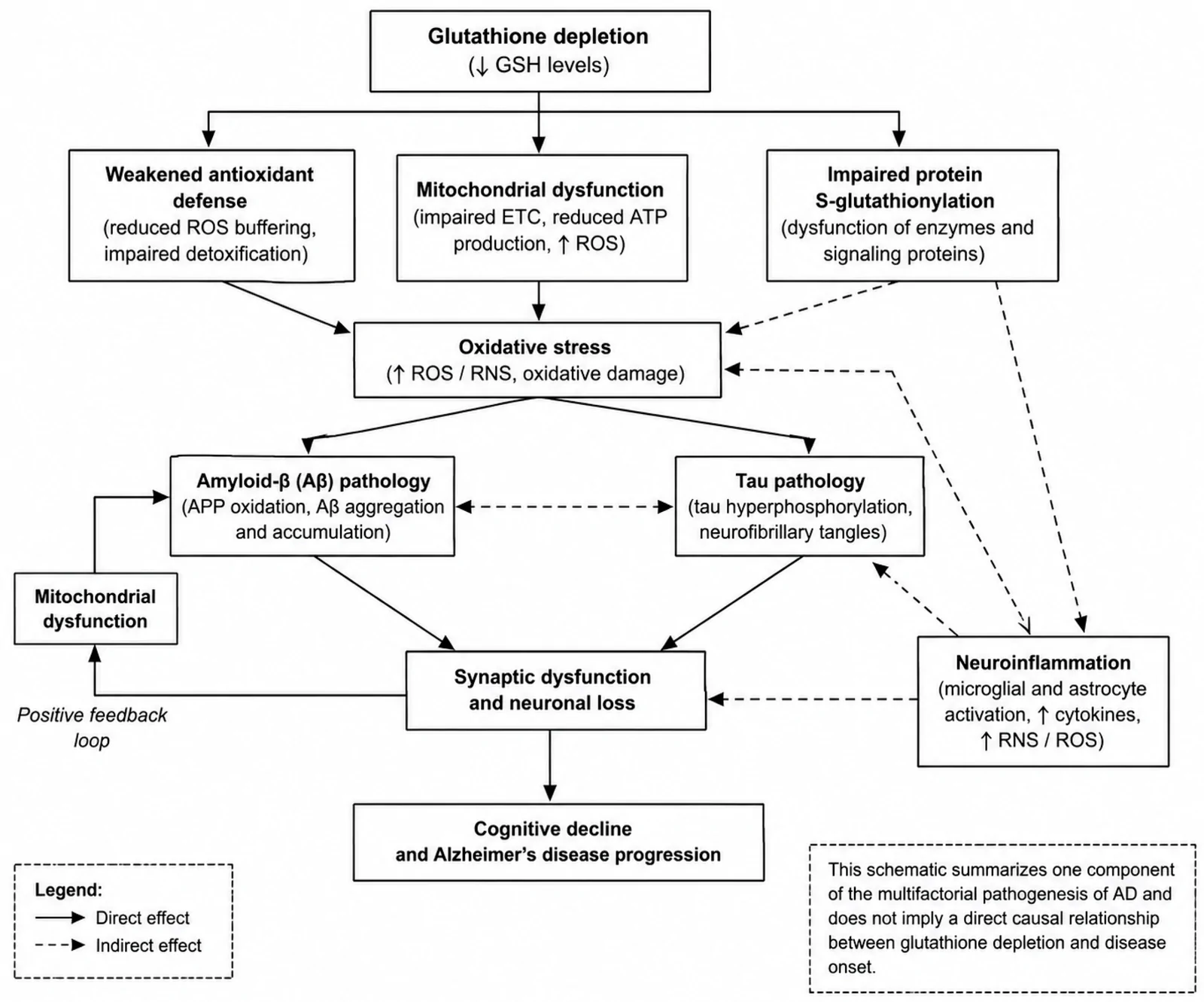
Glutathione dysregulation in Alzheimer’s disease. Abbreviations: Aβ, amyloid-beta; AD, Alzheimer’s disease; APP, amyloid precursor protein; ATP, adenosine triphosphate; ETC, electron transport chain; GSH, reduced glutathione; RNS, reactive nitrogen species; ROS, reactive oxygen species.

The interactions among glutathione depletion, Aβ accumulation, tau pathology, synaptic dysfunction, and progressive neurodegeneration are summarized in Figure 3. Together, these mechanisms indicate that impaired glutathione homeostasis contributes to disease progression within a complex pathogenic network but should not be interpreted as the primary initiating event in AD.

### 4.3. Other Disorders

#### 4.3.1. Huntington’s Disease

Huntington’s disease (HD) is an autosomal dominant neurodegenerative disorder caused by an expanded CAG trinucleotide repeat in the *HTT* gene, resulting in the production of mutant huntingtin (mHTT) [132,133,134]. Progressive accumulation of mHTT disrupts multiple cellular processes, including transcriptional regulation, mitochondrial function, axonal transport, proteostasis, and energy metabolism, ultimately leading to selective degeneration of medium spiny neurons in the striatum [133,134,135]. Although oxidative stress is consistently observed in HD, it is currently regarded as one component of a complex pathogenic network initiated primarily by mutant huntingtin toxicity rather than an independent cause of neuronal degeneration.

A characteristic feature of HD is the profound dysregulation of gene expression induced by mutant huntingtin. Among the affected transcriptional regulators, peroxisome proliferator-activated receptor gamma coactivator 1-alpha (PGC-1α) appears particularly important because it coordinates mitochondrial biogenesis, oxidative metabolism, and antioxidant defence. Impaired PGC-1α activity contributes to defective oxidative phosphorylation, reduced ATP production, and diminished expression of genes involved in cellular antioxidant protection, thereby increasing neuronal susceptibility to metabolic stress [136,137,138].

In addition to transcriptional dysregulation, HD is distinguished by abnormalities in cysteine metabolism. Since cysteine is the rate-limiting precursor for glutathione synthesis, disturbances in the transsulfuration pathway may directly compromise intracellular GSH availability [139,140,141]. An experimental study has demonstrated reduced expression and activity of cystathionine γ-lyase (CSE), leading to impaired endogenous cysteine production and decreased glutathione synthesis [140]. These alterations may further compromise mitochondrial function and increase neuronal vulnerability to oxidative damage. Nevertheless, the precise contribution of impaired transsulfuration to human HD pathogenesis remains incompletely understood [140].

Within this metabolic context, glutathione depletion appears to be largely secondary to mutant huntingtin-induced disturbances in transcriptional regulation and sulfur-containing amino acid metabolism [140,141,142]. Experimental studies have demonstrated reduced intracellular GSH levels, impaired activity of antioxidant enzymes, and increased oxidative damage in cellular and animal models of HD [142,143,144,145]. Similar alterations have also been reported in patient-derived samples, although their magnitude and clinical significance remain variable across studies.

Mutant huntingtin also promotes chronic activation of microglia and astrocytes, resulting in sustained production of pro-inflammatory mediators and reactive oxygen species. These inflammatory responses further aggravate metabolic dysfunction and oxidative injury, contributing to progressive neuronal degeneration [146,147,148,149]. Thus, impaired transcriptional regulation, defective cysteine metabolism, glutathione depletion, and neuroinflammation should be viewed as interconnected downstream consequences of mutant huntingtin toxicity rather than independent pathogenic pathways [136,140,141,148].

Several glutathione-targeted therapeutic approaches have shown encouraging results in experimental models. Administration of NAC, pharmacological activation of the Nrf2 pathway, and interventions aimed at improving mitochondrial bioenergetics reduce oxidative damage and partially preserve neuronal function in preclinical studies [150,151,152,153,154]. However, convincing clinical evidence demonstrating therapeutic efficacy in patients with HD is still lacking [155]. Consequently, restoration of glutathione homeostasis should currently be regarded as an adjunctive strategy that may complement disease-modifying approaches directed at the primary molecular defect.

**Figure 4:**
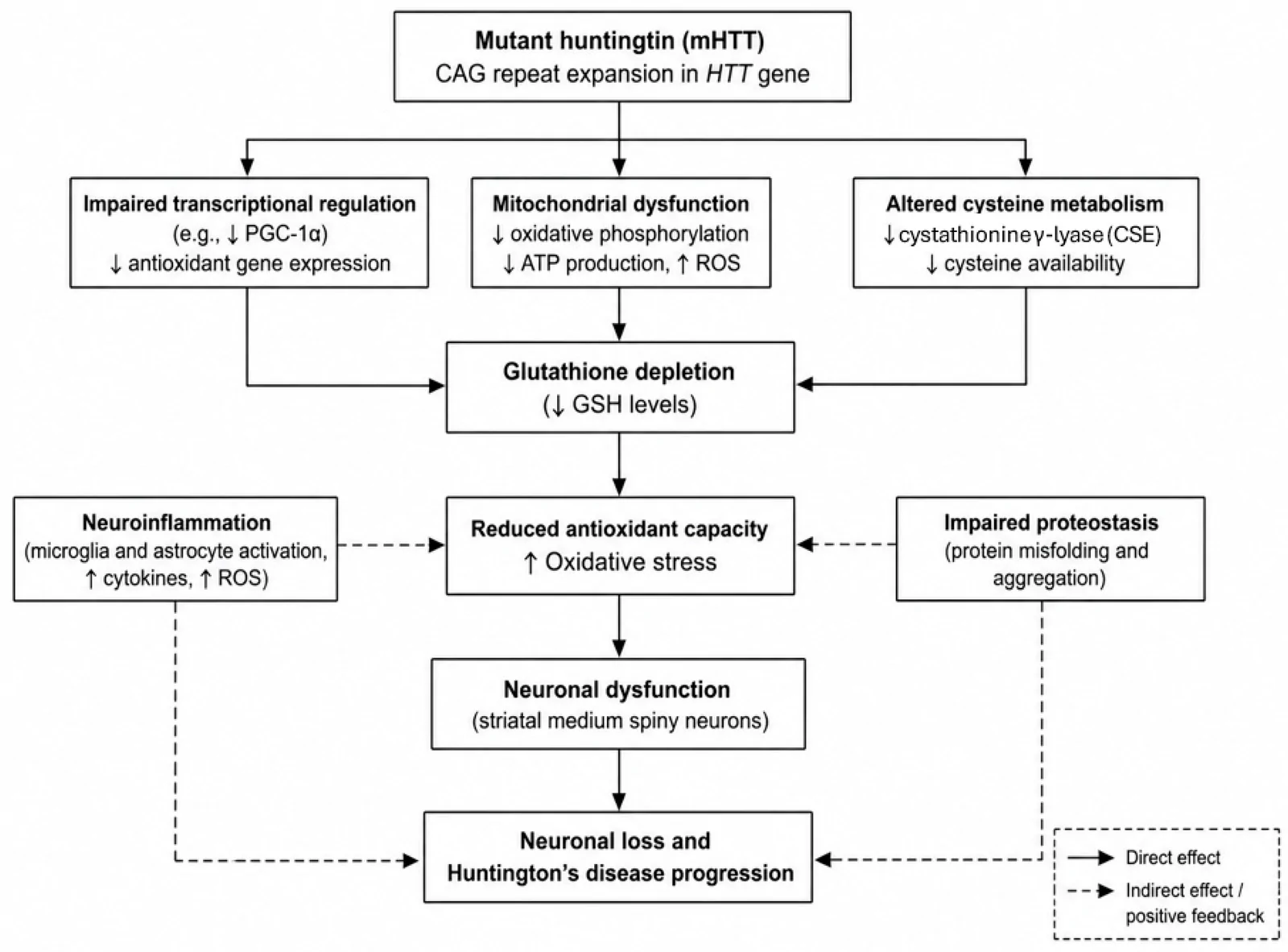
Glutathione dysregulation in Huntington’s disease. Abbreviations: ATP, adenosine triphosphate; CAG, cytosine–adenine–guanine; CSE, cystathionine γ-lyase; GSH, reduced glutathione; HTT, huntingtin; mHTT, mutant huntingtin; PGC-1α, peroxisome proliferator-activated receptor gamma coactivator 1-alpha; ROS, reactive oxygen species.

The relationships among mutant huntingtin, impaired PGC-1α signaling, abnormalities of cysteine metabolism and transsulfuration, glutathione depletion, oxidative stress, and neuroinflammation are summarized in Figure 4.

#### 4.3.2. Multiple Sclerosis

Multiple sclerosis (MS) is a chronic immune-mediated disease of the CNS characterized by inflammatory demyelination, axonal degeneration, and progressive neuroldisability. The disease develops through complex interactions among genetic susceptibility, environmental factors, dysregulated adaptive and innate immune responses, and neurodegenerative processes [156,157,158]. Although oxidative stress contributes to tissue injury, it should be regarded primarily as a downstream consequence of persistent immune activation rather than an isolated pathogenic mechanism [157,158,159,160].

As discussed in Section 3, glutathione contributes to redox homeostasis not only in neurons but also in immune cells, where it regulates cellular activation, cytokine production, and inflammatory signaling [161,162]. Several studies have demonstrated reduced cerebral glutathione concentrations together with increased markers of oxidative damage in the brains, cerebrospinal fluid, and peripheral blood of patients with MS, although the magnitude of these alterations varies according to disease phenotype and methodology [163,164].

These findings suggest that glutathione dysregulation contributes to the inflammatory microenvironment rather than acting as an independent driver of disease.

A distinguishing feature of MS is the central role of immune-cell dysfunction in mediating tissue injury. Activated microglia, infiltrating macrophages, and autoreactive T lymphocytes produce pro-inflammatory cytokines together with reactive oxygen and nitrogen species, thereby promoting demyelination and axonal injury [157,158,159]. In this inflammatory milieu, oligodendrocytes are particularly vulnerable because of the high metabolic demands associated with myelin synthesis and maintenance, combined with their relatively limited antioxidant reserve [165,166].

Beyond protecting mature oligodendrocytes, glutathione may also influence the efficiency of remyelination. Experimental evidence indicates that impaired glutathione-dependent detoxification promotes apoptosis of differentiating oligodendrocytes, thereby limiting endogenous remyelination following inflammatory injury [167]. Consequently, glutathione depletion may contribute not only to increased susceptibility of myelin to immune-mediated damage but also to incomplete remyelination during disease progression.

Unlike the neurodegenerative disorders discussed previously, therapeutic strategies in MS primarily target immune dysregulation. In this context, dimethyl fumarate represents an important example because its clinical efficacy is thought to result from both immunomodulatory activity and activation of the Nrf2 signaling pathway, which enhances endogenous antioxidant defences and supports glutathione homeostasis [168,169,170,171]. Thus, modulation of glutathione metabolism appears to complement, rather than replace, the principal immunological mechanisms underlying disease modification.

Other glutathione-targeted interventions, including NAC supplementation, have shown encouraging effects on biochemical and imaging findings in experimental studies and small clinical trials [172,173,174,175]. However, robust evidence demonstrating sustained clinical benefit remains limited, and larger randomized controlled trials are required to determine whether therapies specifically targeting glutathione metabolism provide additional value beyond established disease-modifying treatments.

**Figure 5:**
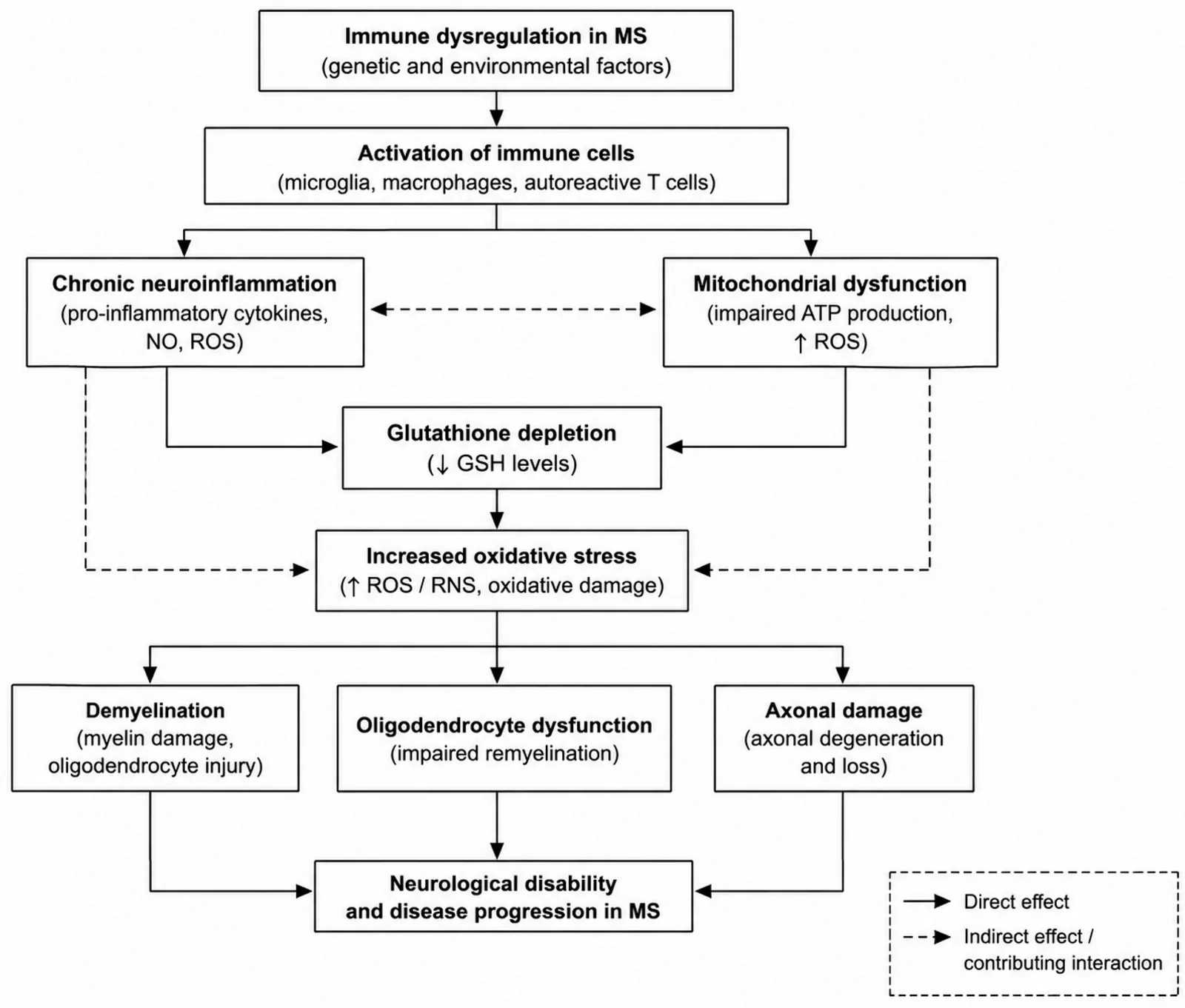
Glutathione dysregulation in multiple sclerosis. Abbreviations: ATP, adenosine triphosphate; GSH, reduced glutathione; MS, multiple sclerosis; NO, nitric oxide; RNS, reactive nitrogen species; ROS, reactive oxygen species.

The interactions among immune-cell activation, macrophage and microglial responses, oligodendrocyte dysfunction, impaired remyelination, glutathione dysregulation, and progressive neurodegeneration are summarized in Figure 5. Together, these mechanisms indicate that glutathione contributes to maintaining both immune and glial homeostasis and should be viewed as an important modulator of inflammatory tissue injury.

#### 4.3.3. Wilson’s Disease

Wilson’s disease (WD) is a rare autosomal recessive disorder caused by pathogenic variants in the *ATP7B* gene, resulting in impaired biliary copper excretion and progressive copper accumulation, primarily in the liver and CNS [176,177]. Although copper overload represents the primary molecular defect, disease severity and clinical manifestations vary considerably among affected individuals, indicating that additional molecular mechanisms influence disease progression [178,179,180].

WD has a unique position at the interface between metabolic and neurodegenerative disorders. Although its primary defect lies in copper metabolism, progressive copper accumulation within the CNS leads to neuronal dysfunction and degeneration, justifying its inclusion in this section.

A characteristic feature of WD is the direct cytotoxic effect of excess intracellular copper. Through redox-active reactions, accumulated copper promotes oxidative modification of proteins, lipids, nucleic acids, and cellular membranes, while simultaneously impairing mitochondrial respiratory chain activity and ATP production [181,182]. Because mitochondria are particularly susceptible to copper toxicity, progressive mitochondrial dysfunction further amplifies metabolic impairment and cellular injury. Nevertheless, oxidative stress should be regarded as a consequence and amplifier of copper toxicity rather than its primary cause [178,183].

Within this context, glutathione serves as one of the principal intracellular defence systems against copper-induced oxidative damage. As discussed in Section 3, glutathione participates in maintaining redox homeostasis, detoxification, and mitochondrial integrity. Depletion of intracellular glutathione may therefore reduce the capacity of hepatocytes and neurons to buffer copper-induced oxidative injury, thereby exacerbating tissue damage without initiating the underlying disease process [178,181,182,183].

An important aspect that distinguishes WD from many other neurodegenerative disorders is its marked phenotypic heterogeneity. Patients carrying identical or closely related *ATP7B* variants often present with remarkably different hepatic, neurological, or psychiatric manifestations [179,180]. Increasing evidence suggests that this variability is influenced, at least in part, by differences in antioxidant defence capacity. Elevated oxidative damage has been associated particularly with neurological and psychiatric involvement, while alterations in antioxidant enzyme activity and lipid peroxidation correlate with disease severity [184].

Genetic variability within antioxidant defence pathways may further contribute to this heterogeneity. Polymorphisms in genes encoding glutathione peroxidase 1 (*GPX1*), superoxide dismutase 2 (*SOD2*), and catalase (*CAT*) have been associated with differences in clinical presentation among patients with WD [185]. These observations support the concept that glutathione-dependent antioxidant systems function primarily as disease-modifying factors that influence tissue susceptibility to copper toxicity rather than determinants of disease initiation.

Current treatment of WD is based on lifelong copper-lowering therapy using chelating agents or zinc salts, which effectively reduce systemic copper burden but do not directly correct oxidative injury [176]. Experimental studies suggest that antioxidant approaches, including NAC, may enhance glutathione-dependent antioxidant defenses and attenuate copper-induced oxidative stress, although NAC may also influence the homeostasis of essential trace elements, including copper and zinc [186]. However, convincing clinical evidence supporting glutathione-based therapies is currently lacking, and no antioxidant intervention has demonstrated disease-modifying efficacy beyond established copper-directed treatment. Consequently, strategies aimed at restoring glutathione homeostasis should currently be regarded as supportive interventions that may complement, but not replace, conventional therapy.

**Figure 6:**
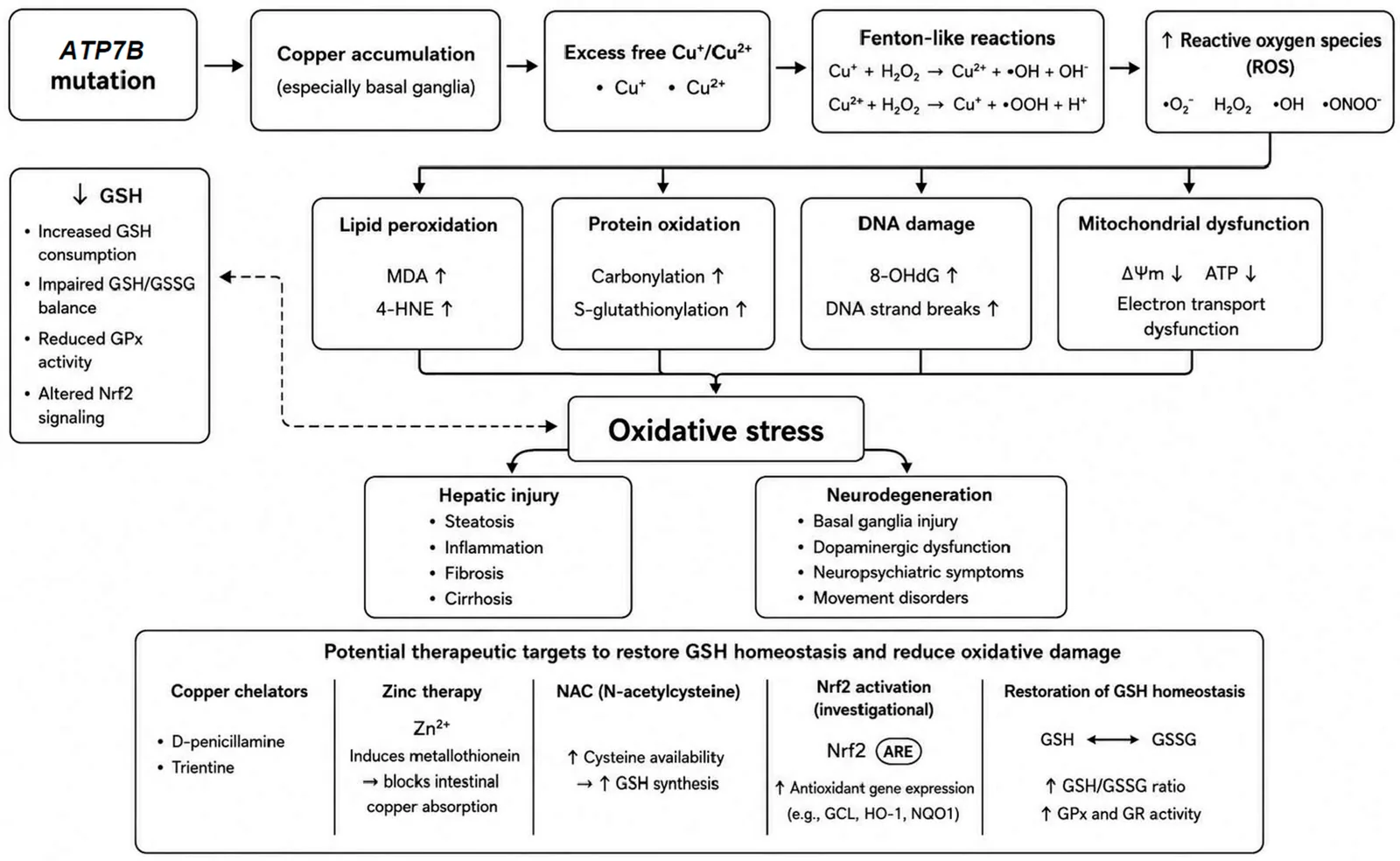
Glutathione dysregulation in Wilson’s disease. Abbreviations: 4-HNE, 4-hydroxynonenal; 8-OHdG, 8-hydroxy-2′-deoxyguanosine; ARE, antioxidant response element; ATP, adenosine triphosphate; ATP7B, ATPase copper transporting beta; Cu, copper; Cu^+^, cuprous ion; Cu^2+^, cupric ion; DNA, deoxyribonucleic acid; GCL, glutamate–cysteine ligase; GPx, glutathione peroxidase; GR, glutathione reductase; GSH, reduced glutathione; GSSG, oxidized glutathione (glutathione disulfide); HO-1, heme oxygenase-1; MDA, malondialdehyde; NAC, N-acetylcysteine; NQO1, NAD(P)H quinone oxidoreductase 1; Nrf2, nuclear factor erythroid 2–related factor 2; ROS, reactive oxygen species; ΔΨm, mitochondrial membrane potential.

The relationships among copper accumulation, mitochondrial dysfunction, glutathione depletion, oxidative injury, clinical heterogeneity, and liver and nervous system damage are summarized in Figure 6. Together, these mechanisms indicate that glutathione primarily modulates cellular susceptibility to copper toxicity, whereas impaired copper transport caused by ATP7B dysfunction remains the initiating event in WD.

## 5. Glutathione in Metabolic Diseases

### 5.1. Type 2 Diabetes

T2D is a multifactorial metabolic disorder characterized by chronic hyperglycemia resulting from insulin resistance and progressive pancreatic β-cell dysfunction [187,188]. Disease progression reflects complex interactions among genetic susceptibility, obesity, glucotoxicity, lipotoxicity, chronic low-grade inflammation, and metabolic stress. Although redox imbalance contributes substantially to these processes, it should be regarded as one component of this multifactorial network rather than the sole determinant of disease progression [187,188,189].

As discussed in Section 3, glutathione is a major component of the endogenous antioxidant defence system and contributes to cellular redox homeostasis in multiple metabolically active tissues. In T2D, however, one of the most distinctive features is the high vulnerability of pancreatic β-cells. Unlike many other cell types, β-cells express relatively low levels of antioxidant enzymes, including catalase and glutathione peroxidase, making them highly dependent on glutathione-mediated antioxidant protection [190,191]. Consequently, even moderate disturbances in glutathione homeostasis may impair β-cell viability and insulin secretory capacity.

Under conditions of chronic glucotoxicity and elevated circulating free fatty acids, progressive metabolic stress overwhelms endogenous antioxidant defenses, contributing to impaired insulin secretion and gradual β-cell loss [192,193,194,195,196]. Rather than acting as an isolated pathogenic mechanism, glutathione depletion appears to reduce the ability of β-cells to adapt to sustained metabolic stress, thereby accelerating functional decline.

**Figure 7:**
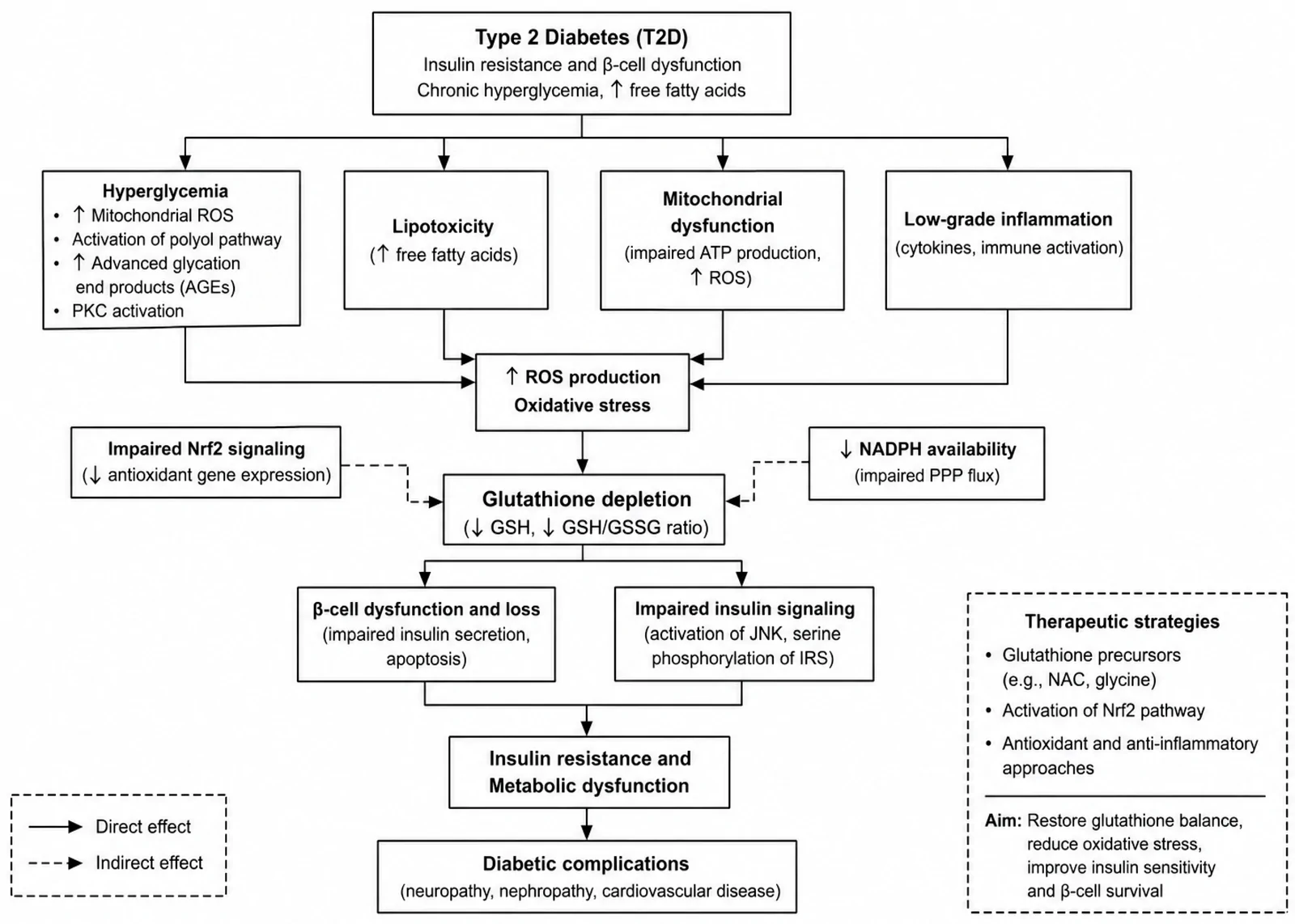
Glutathione dysregulation in type 2 diabetes. Abbreviations: AGEs, advanced glycation end products; ATP, adenosine triphosphate; GSH, reduced glutathione; GSSG, oxidized glutathione (glutathione disulfide); IRS, insulin receptor substrate; JNK, c-Jun N-terminal kinase; NAC, N-acetylcysteine; NADPH, reduced nicotinamide adenine dinucleotide phosphate; Nrf2, nuclear factor erythroid 2–related factor 2; PKC, protein kinase C; PPP, pentose phosphate pathway; ROS, reactive oxygen species; T2D, type 2 diabetes.

Beyond pancreatic islets, glutathione dysregulation also contributes to systemic insulin resistance. Redox imbalance activates stress-responsive kinases, particularly c-Jun N-terminal kinase (JNK), which promotes inhibitory serine phosphorylation of insulin receptor substrate (IRS) proteins in skeletal muscle, liver, and adipose tissue [197,198]. Impaired IRS signaling disrupts downstream insulin signaling, leading to reduced glucose uptake and worsening insulin resistance. These mechanisms interact closely with chronic inflammation, creating a self-reinforcing cycle that promotes metabolic dysfunction [199]. The multifactorial role of glutathione dysregulation in insulin signaling and metabolic homeostasis is summarized in Figure 7.

Experimental evidence also suggests that impaired Nrf2 signaling may contribute to diminished expression of enzymes responsible for glutathione synthesis and recycling under chronic metabolic stress [168,169,200,201]. Pharmacological activation of this pathway improves antioxidant capacity, β-cell survival, and insulin sensitivity in experimental models [202,203]. However, robust clinical evidence demonstrating long-term metabolic benefits remains limited.

Clinical studies consistently demonstrate lower GSH concentrations and a reduced GSH/GSSG ratio in individuals with T2D compared with healthy controls, with more pronounced abnormalities observed in patients who develop diabetic complications, including neuropathy, nephropathy, and cardiovascular disease [20,204]. Nevertheless, these associations do not establish a causal relationship between glutathione depletion and disease progression.

Consequently, increasing attention has been directed toward interventions that restore glutathione homeostasis. Among these, combined glycine and NAC (GlyNAC) supplementation has emerged as one of the most promising approaches because it addresses substrate availability for endogenous glutathione synthesis rather than relying on direct glutathione administration [205,206,207,208]. Preliminary clinical studies have reported improvements in intracellular glutathione concentrations, mitochondrial function, oxidative stress biomarkers, and insulin sensitivity following GlyNAC supplementation. However, the number of available clinical trials remains limited, and larger randomized studies are required to determine whether these metabolic improvements translate into sustained clinical benefits.

### 5.2. Metabolic Dysfunction-Associated Steatotic Liver Disease

Metabolic dysfunction-associated steatotic liver disease (MASLD), formerly referred to as nonalcoholic fatty liver disease (NAFLD), encompasses a spectrum of liver disorders ranging from simple steatosis to metabolic dysfunction-associated steatohepatitis (MASH; formerly nonalcoholic steatohepatitis, NASH), progressive fibrosis, and cirrhosis in the absence of other causes of chronic liver disease [209,210]. Disease progression results from complex interactions among metabolic dysfunction, lipid accumulation, inflammation, and tissue remodeling, with oxidative stress acting as an important contributor rather than an isolated pathogenic mechanism [211,212].

A defining feature of MASLD is hepatocellular lipotoxicity. Excessive accumulation of free fatty acids (FFAs) and other toxic lipid species disrupts hepatocyte metabolism and promotes cellular injury, initiating the transition from simple steatosis to inflammatory liver disease [213,214]. As discussed in Section 3, glutathione represents the principal intracellular antioxidant responsible for maintaining hepatic redox homeostasis [215]. Owing to its important role in hepatic metabolism, glutathione protects hepatocytes against lipid peroxide accumulation and supports detoxification through glutathione peroxidase-dependent reactions and phase II metabolic pathways [139,216].

Under conditions of chronic lipid overload, glutathione consumption progressively exceeds its regeneration, leading to impaired antioxidant capacity and increased susceptibility of hepatocytes to lipotoxic injury [217,218,219,220,221]. Consequently, glutathione depletion appears to amplify the deleterious effects of lipid accumulation rather than initiate disease development.

Beyond hepatocyte injury, progression of MASLD is largely determined by activation of non-parenchymal liver cells. Oxidative lipid metabolites, including malondialdehyde (MDA) and 4-HNE, stimulate Kupffer cells, promoting the release of pro-inflammatory cytokines such as TNF-α and IL-6 [222,223,224,225,226,227]. Persistent inflammatory signaling subsequently activates hepatic stellate cells, leading to extracellular matrix deposition and progressive liver fibrosis [226,227]. Thus, glutathione homeostasis may influence disease progression not only by protecting hepatocytes but also by limiting inflammatory activation and fibrogenic responses within the hepatic microenvironment.

The Nrf2 signaling pathway represents an important adaptive mechanism coordinating antioxidant defence and glutathione metabolism [228,229]. Experimental studies demonstrate that activation of Nrf2 enhances glutathione synthesis, attenuates hepatic inflammation, and limits fibrogenesis in experimental models [229,230]. However, prolonged metabolic stress may impair endogenous Nrf2 responses, and it remains uncertain to what extent pharmacological activation of this pathway translates into clinically meaningful benefit in patients with MASLD [231,232].

Clinical studies have demonstrated reduced hepatic and circulating glutathione concentrations in patients with MASLD, with several reports suggesting associations between impaired glutathione homeostasis and disease severity [221,233]. Nevertheless, available clinical evidence remains heterogeneous because of differences in study populations, disease stage, and methods used to assess glutathione status.

**Figure 8:**
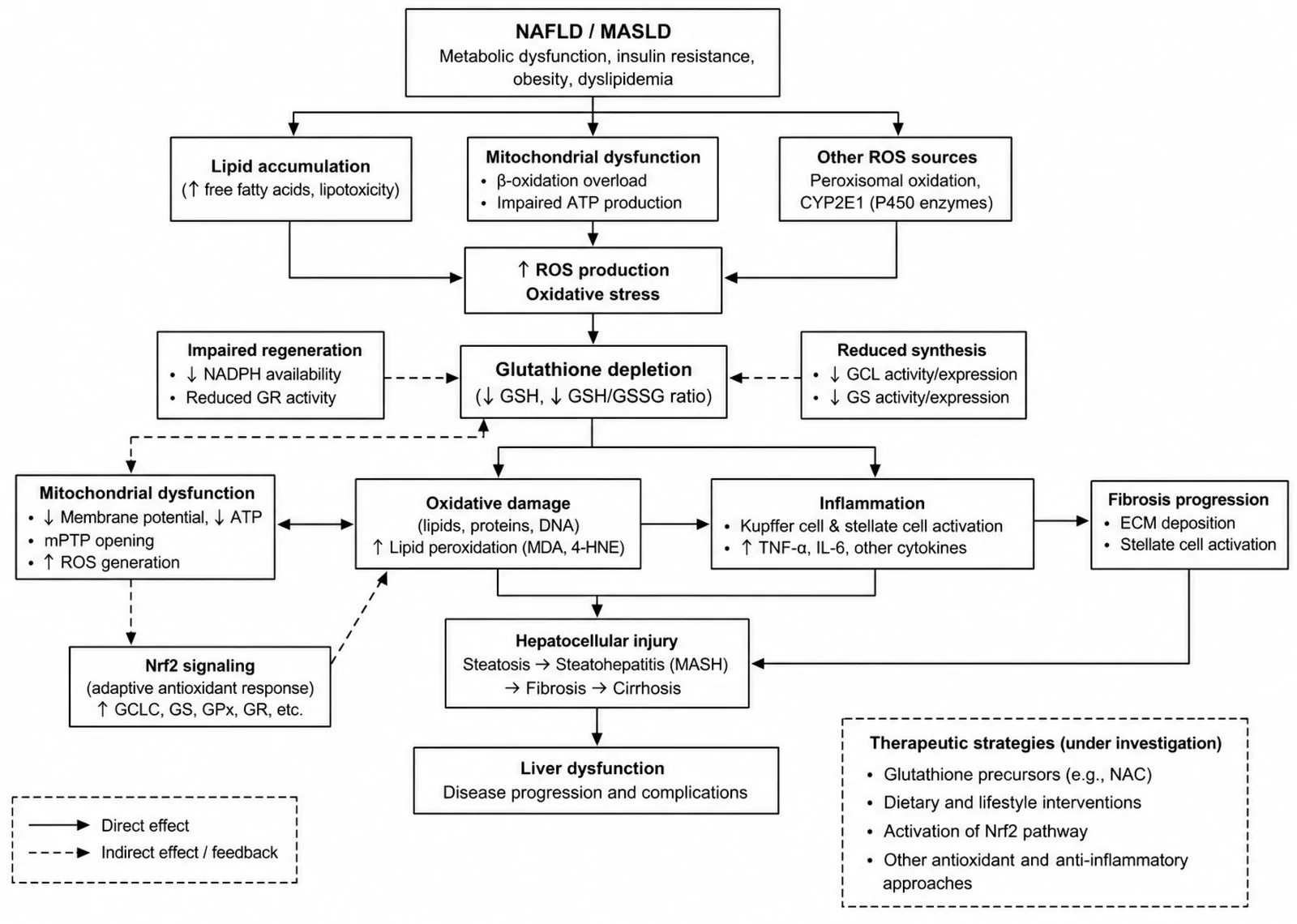
Glutathione dysregulation in metabolic dysfunction-associated steatotic liver disease (MASLD). Abbreviations: 4-HNE, 4-hydroxynonenal; ATP, adenosine triphosphate; CYP2E1, cytochrome P450 family 2 subfamily E member 1; ECM, extracellular matrix; GCL, glutamate–cysteine ligase; GCLC, catalytic subunit of glutamate–cysteine ligase; GPx, glutathione peroxidase; GR, glutathione reductase; GSH, reduced glutathione; GS, glutathione synthetase; GSSG, oxidized glutathione (glutathione disulfide); IL-6, interleukin-6; MASH, metabolic dysfunction-associated steatohepatitis; MASLD, metabolic dysfunction-associated steatotic liver disease; MDA, malondialdehyde; mPTP, mitochondrial permeability transition pore; NAC, N-acetylcysteine; NADPH, reduced nicotinamide adenine dinucleotide phosphate; NAFLD, nonalcoholic fatty liver disease; Nrf2, nuclear factor erythroid 2–related factor 2; ROS, reactive oxygen species; TNF-α, tumor necrosis factor-alpha.

Accordingly, several therapeutic strategies aimed at restoring glutathione homeostasis—including NAC supplementation, dietary interventions, and pharmacological activation of endogenous antioxidant pathways—are currently under investigation [234,235]. Although these approaches have demonstrated beneficial biochemical and histological effects in experimental studies, clinical evidence remains limited and inconsistent. Therefore, glutathione-targeted interventions should currently be regarded as an adjunctive strategy that may complement metabolic and antifibrotic therapies but requires validation in adequately powered randomized clinical trials. The interactions among hepatocellular lipotoxicity, Kupffer cell activation, hepatic stellate cell activation, glutathione depletion, inflammation, and progressive fibrosis are summarized in Figure 8. Together, these mechanisms indicate that impaired glutathione homeostasis acts primarily as a modifier of liver injury and fibrogenesis within the broader metabolic network underlying MASLD rather than as the initiating cause of disease.

### 5.3. Cardiometabolic Integration

Cardiometabolic diseases arise from a complex interplay among metabolic dysregulation, chronic low-grade inflammation, oxidative stress, endothelial dysfunction, and accelerated atherosclerosis [236,237]. Rather than representing distinct pathological entities, obesity, insulin resistance, T2D, MASLD, and cardiovascular disease share multiple molecular mechanisms. As discussed in previous sections, glutathione is a major regulator of intracellular redox homeostasis and contributes to the integration of these interconnected processes [12,216].

A hallmark of cardiometabolic disease is persistent low-grade inflammation (“metaflammation”), which develops in parallel with chronic redox imbalance [238,239]. Nutrient excess promotes mitochondrial ROS production and activation of redox-sensitive signaling pathways, including NF-κB and JNK, thereby increasing the production of pro-inflammatory cytokines such as TNF-α and IL-6 [240,241,242]. Progressive depletion of GSH further weakens antioxidant defences, facilitating sustained oxidative and inflammatory signaling.

Beyond its antioxidant function, disturbances in glutathione homeostasis influence several pathways involved in insulin signaling and vascular function. Oxidative stress impairs insulin receptor signaling through activation of stress-responsive kinases and abnormal serine phosphorylation of IRS proteins, contributing to systemic insulin resistance [241,243,244]. In parallel, altered protein S-glutathionylation may modify the activity of signaling proteins involved in glucose metabolism and endothelial function, although the precise contribution of these mechanisms in human cardiometabolic disease remains incompletely understood [245,246].

Endothelial dysfunction represents another important consequence of impaired redox homeostasis. Reduced GSH availability and excessive ROS production decrease nitric oxide (NO) bioavailability through endothelial nitric oxide synthase (eNOS) uncoupling, promoting vasoconstriction, inflammation, platelet activation, and the expression of adhesion molecules that facilitate leukocyte recruitment to the vascular wall [247,248,249,250]. Oxidative modification of low-density lipoproteins further accelerates foam cell formation and atherosclerotic plaque development, linking metabolic abnormalities with cardiovascular complications [251,252,253,254,255,256,257].

The Nrf2 signaling pathway coordinates many endogenous antioxidant responses, including glutathione synthesis and recycling [258,259]. Experimental studies demonstrate that activation of Nrf2 improves endothelial function, attenuates inflammation, and restores glutathione homeostasis. However, whether pharmacological modulation of this pathway translates into sustained cardiovascular benefit in patients with cardiometabolic disease remains to be established in large clinical trials [260,261,262].

Clinical studies consistently report associations between reduced glutathione levels, insulin resistance, endothelial dysfunction, metabolic syndrome, and increased cardiovascular risk [263,264]. Nevertheless, current evidence is largely observational and does not establish a direct causal relationship between glutathione depletion and cardiometabolic disease progression.

Collectively, these findings indicate that glutathione functions as an important modulator linking disturbed redox homeostasis, inflammation, metabolic dysfunction, and vascular injury. Accordingly, therapeutic strategies aimed at restoring glutathione homeostasis may complement established approaches targeting metabolic risk factors, although further clinical studies are required to determine their long-term efficacy and clinical relevance [265,266].

## 6. Summary: Glutathione in Neurodegenerative and Metabolic Diseases

Glutathione is an essential component of the intracellular antioxidant system and plays a central role in maintaining cellular redox balance. As discussed in the preceding sections, disturbances in glutathione metabolism are found in many neurodegenerative and metabolic diseases. Nevertheless, contemporary scientific data demonstrate that glutathione homeostasis impairment can be considered as one element of a complex network of mechanisms such as mitochondrial dysfunction, inflammation, and metabolic dysregulation involved in disease development.

Despite their different etiologies, these diseases share several molecular mechanisms. In neurodegenerative diseases (PD, AD, HD, MS, WD), glutathione deficiency is correlated with redox imbalance, mitochondrial dysfunction, neuroinflammation, and neuronal damage. In metabolic diseases (T2D, MASLD), disturbances in glutathione metabolism are related to insulin resistance, β-cell dysfunction, lipotoxicity, endothelial dysfunction, and chronic inflammation.

One of the main concepts based on modern research is the interaction between glutathione metabolism and redox-dependent signaling pathways. The changes in the Nrf2 pathway, mitochondrial glutathione homeostasis, and protein S-glutathionylation may play a key role in cellular response to oxidative stress and disease development.

However, the relative importance of these pathways varies depending on the particular disease and is not fully understood. Importantly, most currently available evidence demonstrates associations between glutathione dysregulation and disease progression rather than establishing direct causal relationships.

Based on the data discussed above, one can conclude that glutathione homeostasis restoration can serve as an appropriate biological target. Nonetheless, although numerous experimental studies have shown beneficial effects of restoring glutathione homeostasis, clinical evidence remains limited, and findings have been inconsistent in some disease settings. Thus, additional clinical trials are needed to prove the efficacy of such an approach. Future studies should focus on disease-specific redox signatures, tissue-specific glutathione regulation, biomarker-guided patient stratification, and randomized clinical trials evaluating glutathione-targeted interventions.

Main molecular events associated with glutathione homeostasis disorder are listed in Table 3, while major alterations found in neurodegenerative and metabolic disorders are shown in Table 4.

## 7. Translational Perspectives

### 7.1. Dietary and Nutritional Modulation

Dietary and nutritional strategies aimed at supporting endogenous glutathione homeostasis have attracted considerable interest as potential approaches to mitigating oxidative stress in chronic diseases. Rather than directly replacing intracellular GSH, most nutritional interventions aim to enhance its synthesis, improve antioxidant capacity, or modulate redox-sensitive signaling pathways. Although numerous experimental studies have reported beneficial effects, clinical evidence remains heterogeneous and should be interpreted with caution.

One of the best-studied interventions is NAC, a stable cysteine precursor that provides the rate-limiting substrate for GSH synthesis. Because cysteine availability is a major determinant of intracellular GSH levels, NAC supplementation enhances endogenous GSH synthesis (particularly under conditions of redox imbalance) [267,268]. In addition to serving as a glutathione precursor, NAC exerts direct antioxidant effects through ROS scavenging and modulation of redox-sensitive signaling pathways, including NF-κB and mitogen-activated protein kinase (MAPK) signaling [269,270].

Experimental studies have consistently demonstrated that NAC attenuates oxidative damage, reduces inflammatory responses, and improves mitochondrial function in models of neurodegenerative, metabolic, and cardiovascular diseases [269,270]. Clinical studies have reported improvements in selected biomarkers of oxidative stress and inflammation. Meta-analyses indicate that NAC supplementation may reduce lipid peroxidation, improve total antioxidant capacity, and increase glutathione availability in some patient populations [271,272]. However, considerable heterogeneity exists regarding study populations, treatment duration, dosage, and clinical endpoints. Consequently, current evidence is insufficient to recommend routine NAC supplementation as standard therapy for neurodegenerative or metabolic diseases [273].

Beyond pharmacological supplementation, adequate dietary intake of glutathione precursors remains essential for maintaining endogenous antioxidant capacity. Cysteine, glycine, and glutamate provide the substrates required for GSH biosynthesis, while micronutrients such as selenium and riboflavin support the activity of glutathione-dependent enzymes, including glutathione peroxidase and glutathione reductase [274,275]. Nutritional deficiencies affecting these components may impair glutathione metabolism and increase susceptibility to disturbed redox homeostasis.

Certain dietary components may stimulate endogenous antioxidant responses. Broccoli and Brussels sprouts are cruciferous vegetables rich in sulforaphane, which can activate the Nrf2 pathway and increase the expression of genes involved in glutathione synthesis and detoxification [276,277]. Similarly, dietary patterns rich in fruits, vegetables, whole grains, and unsaturated fatty acids, particularly the Mediterranean diet, have been associated with improved oxidative stress profiles and better metabolic health. Although these effects may partly reflect improved glutathione homeostasis, they are likely mediated through multiple complementary mechanisms rather than glutathione alone [278,279].

Growing evidence also suggests that the gut microbiota influences glutathione metabolism through regulation of amino acid availability, microbial metabolite production, and host redox signaling [280,281]. However, the mechanisms underlying these interactions remain incompletely understood, and their therapeutic implications require further investigation.

Future studies should address the marked interindividual variability in response to nutritional interventions. Genetic background, age, dietary habits, gut microbiota composition, baseline nutritional status, and disease stage all influence glutathione metabolism and may determine treatment efficacy [27,174,175]. These findings support the development of precision nutrition strategies aimed at identifying patients most likely to benefit from interventions targeting glutathione homeostasis.

In summary, dietary interventions, provision of glutathione precursors, and manipulation of the body’s endogenous antioxidant system through nutritional therapy can provide beneficial complementary therapies in achieving redox balance. Nonetheless, such approaches should currently be considered as supportive rather than curative, with further randomized controlled clinical trials required to confirm their effectiveness [189].

Despite encouraging experimental evidence, direct glutathione supplementation has not consistently translated into clinical benefit. Native glutathione exhibits limited oral bioavailability due to extensive gastrointestinal degradation and poor cellular uptake, making sustained elevation of intracellular GSH difficult to achieve. Consequently, current strategies increasingly focus on glutathione precursors, such as NAC and GlyNAC, or on stimulation of endogenous glutathione synthesis through activation of Nrf2-dependent pathways. Furthermore, substantial interindividual variability in glutathione metabolism, influenced by genetic background, age, nutritional status, gut microbiota, and disease stage, may explain the inconsistent outcomes observed across clinical trials. These observations support a precision medicine approach in which redox-targeted interventions are tailored to patient-specific characteristics.

### 7.2. Pharmacological Activators

Pharmacological modulation of glutathione metabolism has emerged as a promising strategy for restoring redox homeostasis in disorders characterized by oxidative stress. In contrast to nutritional interventions, pharmacological approaches target specific components of glutathione metabolism or its upstream regulatory pathways, including GSH synthesis, recycling, intracellular transport, and Nrf2 signaling. Although many of these strategies have shown encouraging results in experimental models, their clinical translation remains limited [282,283,284].

One of the most extensively researched pathways is the Nrf2 pathway, which modulates the expression of several antioxidant and cytoprotective genes. Under basal conditions, Nrf2 remains in the cytoplasm bound to Keap1 and undergoes continuous proteasomal degradation [285]. Electrophilic compounds such as dimethyl fumarate (DMF) disrupt the Keap1-Nrf2 interaction by modifying reactive cysteine residues within Keap1, allowing Nrf2 to accumulate and translocate to the nucleus [286,287]. Subsequent activation of ARE-dependent transcription enhances the expression of genes involved in glutathione synthesis, recycling, and utilization, including *GCL*, *GS*, *GR*, *GPX*, and glutathione S-transferase (*GST*) [286,287].

Experimental studies have consistently demonstrated that activation of the Nrf2 pathway increases intracellular GSH levels, attenuates oxidative stress, and suppresses inflammatory signaling through modulation of redox-sensitive pathways, including NF-κB [288,289]. Among currently available therapies, DMF is a well-known example of a pharmacologically induced activator of the transcription factor Nrf2, and it has been found to be effective for the treatment of multiple sclerosis. However, more than one mechanism is involved in its mode of action [290].

Beyond Nrf2, several pharmacological strategies aim to modulate enzymes directly involved in glutathione metabolism. Enhancement of GCL activity may increase endogenous GSH synthesis, particularly during disturbed redox homeostasis [291,292]. Likewise, preservation of glutathione recycling through maintenance of GR activity and adequate NADPH availability may improve intracellular redox balance [293]. On the other hand, GPx or GST inhibition has been mainly investigated in oncology to enhance redox imbalance specifically within tumor cells, highlighting the disease-specific applicability of interventions targeting glutathione metabolis [294,295].

Additional experimental approaches target glutathione degradation and intracellular distribution. Inhibition of γ-glutamyl transpeptidase (GGT), the enzyme responsible for extracellular glutathione catabolism, has been proposed as a means of preserving extracellular glutathione availability [296]. Equally, much attention has been paid to the significance of mGSH since it is important for maintaining mitochondrial integrity as well as protection against oxidative stress. Pharmacological approaches aimed at preserving and restoring mitochondrial glutathione transport could prove more specific than simply increasing total cellular glutathione levels [24,297].

Another area of active investigation involves glutathione mimetics and prodrugs, which aim to overcome the limited bioavailability and poor cellular uptake of native glutathione [298,299]. Indeed, although a number of compounds showed favorable pharmacokinetic properties and antioxidant activity in laboratory conditions, adequate evidence of their effectiveness still does not exist.

An additional challenge is the identification of patient populations most likely to benefit from redox-targeted therapies. Oxidative stress is highly dynamic and varies according to disease stage, comorbidities, genetic factors, and baseline antioxidant capacity. Consequently, indiscriminate antioxidant supplementation may not produce uniform clinical effects, emphasizing the need for biomarker-guided therapeutic strategies.

Pharmacological activation of antioxidant responses should be considered with great care. Reactive oxygen species are involved in regular intracellular signaling and adaptation processes. Thus, their excessive inhibition may lead to unpredictable biological consequences.

In general, pharmacologic manipulation of glutathione pathways seems to be a very promising therapeutic area. Although some methods have shown significant promise both in laboratory and in some clinical studies, more randomized clinical trials are needed to prove their long-term effectiveness, safety, proper indications, and their appropriate indications and place in current treatment strategies.

Future progress will likely depend not only on the development of more effective glutathione-modulating compounds but also on improved patient stratification using biomarkers of redox status, glutathione metabolism, and disturbed redox homeostasis. Such precision medicine approaches may help identify individuals most likely to benefit from glutathione-targeted interventions while avoiding ineffective treatment in patients without demonstrable redox imbalance.

### 7.3. Lifestyle Interventions

Non-pharmacological approaches such as lifestyle modification may provide complementary strategies for maintaining glutathione homeostasis. In contrast to pharmacological interventions, lifestyle interventions impact several physiological systems at once, for example, mitochondria, metabolism, immune system, and neuroendocrine signaling. Direct evidence linking lifestyle modification with glutathione metabolism remains limited, but there are some findings indicating the beneficial effects of lifestyle modification on oxidative stress and antioxidant potential [300,301,302,303].

Regular physical activity is among the best-characterized lifestyle factors influencing redox homeostasis. Exercise leads to acute elevation of ROS levels, which elicits hormesis through induction of cellular stress and not through continuous redox imbalance. The processes include induction of Nrf2 pathway activation and expression of antioxidants that aid in GSH biosynthesis and metabolism [300,304,305,306]. The magnitude of these effects depends on exercise intensity, duration, training status, age, and underlying disease.

Sleep quality and circadian regulation also contribute to the maintenance of redox homeostasis. Experimental studies indicate that sleep deprivation and chronic circadian disruption increase oxidative stress while reducing glutathione availability in both the CNS and peripheral tissues [293,301,302,307]. Conversely, adequate sleep appears to facilitate restoration of the GSH/GSSG ratio, support mitochondrial function, and promote recovery of antioxidant defences. Nonetheless, most data originate from experimental models, and additional clinical studies are required to establish impact of sleep interventions on glutathione metabolism.

The effects of these lifestyle modifications should be considered collectively rather than in isolation. Regular physical activity, adequate sleep, a balanced diet, maintenance of a healthy body weight, smoking cessation, and avoidance of excessive alcohol consumption may all contribute to reducing redox imbalance and improving metabolic health. These effects are mediated through multiple interconnected mechanisms, of which improved glutathione homeostasis is only one.

Lifestyle modification is generally a safe and broadly applicable approach to supporting endogenous antioxidant defences. Even though these measures cannot be used as glutathione-specific treatment now, they can be combined with pharmacological and dietary approaches to reduce oxidative stress and improve metabolic resilience.

Table 5 summarizes the main dietary, pharmacological, and lifestyle strategies aimed at modulating glutathione homeostasis, together with their principal mechanisms of action, current level of evidence, and major limitations.

## 8. Future Research Directions

Though much knowledge has been gained about glutathione physiology, there are still many questions that need to be addressed. The key areas for future investigations include the elucidation of the cause-and-effect relationship between glutathione dysfunction and disease development, identification of biomarkers that would help assess redox balance, and the application of newly acquired information for developing treatments.

### 8.1. Dynamic Assessment of Glutathione Homeostasis

Most current knowledge regarding glutathione status is derived from static measurements of total GSH or the GSH/GSSG ratio obtained from blood or tissue samples. However, glutathione homeostasis is a highly dynamic process that varies across tissues, cell types, and disease stages. Future research should therefore prioritize longitudinal assessment of glutathione metabolism using standardized analytical methods and clinically validated biomarkers. Promising candidates include the GSH/GSSG ratio, protein *S*-glutathionylation, glutathione-related enzyme activities, and mitochondrial glutathione (mGSH) status. Furthermore, the development of non-invasive approaches, such as magnetic resonance spectroscopy (MRS) for in vivo brain glutathione assessment, together with advances in redox metabolomics, may improve the monitoring of temporal changes in oxidative stress, facilitate evaluation of therapeutic responses, and support the implementation of precision medicine strategies.

### 8.2. Mitochondrial Glutathione, Aging, and Epigenetic Regulation

Growing evidence indicates that mGSH represents a distinct intracellular glutathione pool that plays an important role in maintaining mitochondrial redox homeostasis and protecting mitochondria from oxidative damage [24]. Further studies are needed to elucidate the mechanisms governing mGSH transport, compartmentalization, and turnover, particularly in aging and in neurodegenerative and metabolic diseases [24,308]. Increasing evidence also indicates that glutathione metabolism is interconnected with epigenetic regulation. DNA methylation, histone modifications, and non-coding RNAs can influence the expression of genes involved in redox regulation and glutathione metabolism, while cellular metabolic and redox states may in turn affect epigenetic processes [309,310,311]. These reciprocal interactions may become particularly relevant during aging, when progressive alterations in mitochondrial function, redox homeostasis, and epigenetic regulation occur [312]. Further investigation of the interplay between glutathione metabolism, mitochondrial dysfunction, and epigenetic regulation may therefore provide new insights into mechanisms of aging and age-related diseases and may identify potential targets for therapeutic intervention [24,312].

### 8.3. Precision Redox Medicine

One of the major challenges in glutathione research is the marked heterogeneity observed among patients with similar clinical diagnoses. Future studies should investigate how genetic variation, environmental exposures, nutritional status, gut microbiota composition, aging, and comorbidities influence glutathione metabolism and treatment response. Such knowledge may facilitate the development of precision medicine approaches based on individual redox phenotypes and enable identification of patients most likely to benefit from these interventions.

### 8.4. Multi-Omics and Systems Biology

The rapid progress in the field of transcriptomics, proteomics, metabolomics, lipidomics, and single-cell sequencing creates unique possibilities to study the biology of glutathione on various regulatory levels. The use of such data together with computational modeling and artificial intelligence in combination with systems biology will allow for better comprehension of redox regulation networks, new biomarker discovery and target validation. The use of multi-omics approaches combined with clinical cohorts and experimental studies will be crucial for the application of basic science in clinical practice.

In general, future studies concerning glutathione should move beyond association toward establishing causality in order to clarify and validate its role in disease prevention and therapy. The use of such an approach will be crucial to elucidate the role of glutathione-targeted interventions in neurodegeneration and metabolic disorders.

The major priorities for future glutathione research are summarized in Table 6.

### 8.5. Future Directions in Translational Glutathione Research

The main challenge for translating glutathione biology into clinical practice is to determine when and in which disease contexts modulation of glutathione homeostasis is likely to provide meaningful clinical benefit. Rather than treating glutathione dysregulation as a universal therapeutic target, future research should focus on establishing causal relationships between altered glutathione metabolism and disease-specific outcomes.

Translational studies should therefore connect mechanistic findings with clinically relevant endpoints and determine whether changes in glutathione status are predictive of treatment response or merely reflect disease-associated redox alterations. This will require adequately powered, well-designed clinical trials and standardized assessment of glutathione-related biomarkers across defined patient populations.

Ultimately, successful translation will depend on integrating mechanistic, molecular, and clinical evidence to identify interventions that complement established disease-specific therapies rather than replacing them.

## 9. Conclusions

Glutathione is a central component of the intracellular antioxidant defense system and an important regulator of cellular redox homeostasis. Beyond its role in protecting against oxidative stress, glutathione contributes to mitochondrial function, redox-sensitive signaling, immune regulation, and cellular adaptation to metabolic and environmental stress.

The evidence reviewed in this article indicates that disturbances in glutathione homeostasis are associated with neurodegenerative and metabolic diseases, where they interact with mitochondrial dysfunction, inflammation, metabolic abnormalities, and altered cellular signaling. However, the contribution of glutathione dysregulation varies considerably according to disease type, stage, tissue context, and other pathological processes. Glutathione abnormalities should therefore be regarded as one component of a complex and multifactorial pathogenic network rather than as an isolated or universal driver of disease.

Although substantial progress has been made in understanding the molecular regulation and biological functions of glutathione, important questions remain regarding the causal significance of glutathione depletion in disease and the clinical utility of redox-related biomarkers. Experimental studies and some clinical trials suggest that nutritional, lifestyle, and pharmacological interventions targeting glutathione homeostasis may enhance antioxidant defences in selected clinical contexts; however, evidence for sustained and clinically meaningful long-term benefits remains limited.

As this is a narrative review, the conclusions should be interpreted in the context of the heterogeneous nature of the available evidence. Further progress will require continued mechanistic and translational research, together with adequately powered clinical studies, to determine when and in which disease contexts modulation of glutathione homeostasis may provide meaningful therapeutic benefit.

## Figures and Tables

**Table 1:** Key Enzymes and Processes in Glutathione Metabolism.

Process	Enzyme	Function	Regulation	Clinical Relevance
GSH synthesis	GCL	Rate-limiting step in GSH synthesis	Nrf2 activation; cysteine availability; feedback inhibition by GSH	Reduced activity → oxidative stress
GSH synthesis	GS	Converts γ-glutamylcysteine to GSH	Glycine availability; ATP availability	Deficiency linked to metabolic disorders
GSH recycling	GR	Reduces GSSG → GSH (NADPH-dependent)	NADPH availability	Impaired in oxidative stress conditions
Detoxification	GPx	Reduces H_2_O_2_ and lipid peroxides	Selenium-dependent	Key in neuroprotection
Conjugation	GSTs	Detoxifies xenobiotics	Induced by oxidative stress	Important in liver diseases and cancer
Degradation	GGT	Breaks down extracellular GSH	Tissue-specific	Elevated in liver disease; biomarker of hepatobiliary disease and altered glutathione turnover

Abbreviations: GSH, glutathione (reduced form); GSSG, glutathione disulfide (oxidized form); GCL, glutamate–cysteine ligase; GS, glutathione synthetase; GR, glutathione reductase; GPx, glutathione peroxidase; GST, glutathione S-transferase; GGT, γ-glutamyl transpeptidase; H_2_O_2_, hydrogen peroxide; NADPH, nicotinamide adenine dinucleotide phosphate (reduced form); Nrf2, nuclear factor erythroid 2–related factor 2.

**Table 2:** Tissue-specific distribution of glutathione and major determinants of oxidative vulnerability.

Tissue	Relative GSH Level	Major Determinants of Oxidative Vulnerability	Principal Physiological Role of GSH
Liver	Very high	Intensive xenobiotic metabolism, ROS generated during detoxification	Detoxification, maintenance of redox homeostasis, conjugation reactions
Kidney	High	High metabolic activity, active tubular transport, exposure to xenobiotics	Protection against oxidative injury, maintenance of tubular function
Lung	High (epithelial lining fluid)	Continuous exposure to environmental oxidants and pollutants	Barrier protection, detoxification of inhaled oxidants
Brain	Moderate	High oxygen consumption, abundant polyunsaturated lipids, limited antioxidant reserve	Protection of neurons from oxidative damage, maintenance of mitochondrial function and neurotransmission
Heart	Moderate–high	Continuous mitochondrial ATP production and oxygen utilization	Preservation of mitochondrial bioenergetics and contractile function
Skeletal muscle	Moderate–high	Exercise-induced ROS generation, high mitochondrial content	Maintenance of redox balance during contraction and recovery
Immune cells	Dynamic	Respiratory burst during immune activation	Regulation of immune-cell activation, proliferation, and cytokine production
Vascular endothelium	Moderate	Shear stress, inflammatory mediators, nitric oxide metabolism	Preservation of endothelial integrity and vascular homeostasis

Abbreviations: GSH, glutathione; ROS, reactive oxygen species.

**Table 3:** Glutathione dysregulation in major neurodegenerative diseases: molecular alterations, pathogenic significance, and current therapeutic evidence.

Disease	Major Glutathione-Related Alterations	Contribution to Disease Pathogenesis	Current Therapeutic Evidence
PD	Early depletion of GSH in the substantia nigra, impaired mitochondrial antioxidant defense, altered redox signaling	Increased susceptibility to oxidative damage, mitochondrial dysfunction, interaction with α-synuclein aggregation and neuroinflammation	N-acetylcysteine and Nrf2 activators show encouraging preclinical results; clinical evidence remains limited and disease-modifying efficacy has not been established.
AD	Reduced GSH concentrations, impaired antioxidant capacity, dysregulated protein S-glutathionylation	Enhanced oxidative stress, mitochondrial dysfunction, amyloid-β and tau pathology within a multifactorial pathogenic network	Experimental studies support antioxidant effects, but convincing clinical evidence for glutathione-targeted therapies is lacking.
HD	Reduced GSH synthesis, altered cysteine metabolism, impaired antioxidant enzyme activity	Increased oxidative stress secondary to mutant huntingtin-mediated mitochondrial and metabolic dysfunction	Preclinical studies demonstrate potential benefit of NAC and Nrf2 activation; no clinically proven disease-modifying therapy is available.
MS	Decreased GSH in CNS and peripheral tissues, impaired antioxidant defense	Amplifies immune-mediated oxidative injury, mitochondrial dysfunction, and demyelination	Dimethyl fumarate indirectly enhances endogenous antioxidant responses through Nrf2 activation; evidence for direct glutathione-targeted interventions remains limited.
WD	Copper-induced depletion of glutathione, impaired antioxidant defense	Potentiates copper-mediated oxidative injury and contributes to clinical heterogeneity rather than initiating disease	Copper-lowering therapy remains the standard of care; antioxidant strategies are investigational and require further clinical validation.

Abbreviations: AD, Alzheimer’s disease; CNS, central nervous system; GSH, reduced glutathione; HD, Huntington’s disease; MS, multiple sclerosis; NAC, N-acetylcysteine; Nrf2, nuclear factor erythroid 2-related factor 2; PD, Parkinson’s disease; WD, Wilson’s disease; the table summarizes the evidence discussed in the corresponding sections of the manuscript and is based on the references cited therein.

**Table 4:** Glutathione dysregulation in major metabolic diseases: alterations in redox homeostasis, pathophysiological significance, and current therapeutic evidence.

Disease	Major Glutathione-Related Alterations	Pathophysiological Significance	Current Therapeutic Evidence
Metabolic dysfunction-associated steatotic liver disease (MASLD)	Decreased hepatic GSH, mitochondrial oxidative stress, impaired detoxification	Promotes lipid peroxidation, inflammation, and progression toward steatohepatitis	Lifestyle intervention remains first-line therapy; antioxidant approaches show variable results and require larger clinical trials.
Obesity	Increased GSH utilization, chronic oxidative stress, impaired antioxidant defense	Contributes to adipose tissue inflammation, mitochondrial dysfunction, and metabolic dysregulation	Weight reduction, exercise, and dietary interventions improve redox status; evidence for direct glutathione supplementation remains inconclusive.
Type 2 diabetes mellitus	Reduced intracellular GSH, impaired glutathione recycling, chronic oxidative stress	Promotes β-cell dysfunction, insulin resistance, endothelial injury, and diabetic complications	NAC and GlyNAC demonstrate promising metabolic effects in early studies, but robust long-term clinical evidence is still lacking.
Cardiovascular disease	Reduced vascular antioxidant capacity, endothelial oxidative stress	Contributes to endothelial dysfunction, inflammation, and atherosclerotic progression	Antioxidant strategies have shown inconsistent clinical benefit despite encouraging experimental findings.

Abbreviations: GSH, reduced glutathione; GlyNAC, glycine plus N-acetylcysteine; MASLD, metabolic dysfunction-associated steatotic liver disease; NAC, N-acetylcysteine; the table summarizes the evidence discussed in the corresponding sections of the manuscript and is based on the references cited therein.

**Table 5:** Current therapeutic strategies targeting glutathione homeostasis: mechanisms of action, level of evidence, and major limitations.

Strategy	Principal Mechanism	Current Evidence	Major Limitations
N-acetylcysteine (NAC)	Provides cysteine for endogenous GSH synthesis	Extensive preclinical evidence; small clinical studies report improvements in selected biomarkers	Variable bioavailability, heterogeneous patient populations, limited evidence for disease modification
GlyNAC (glycine + NAC)	Restores precursor availability for GSH synthesis	Promising early clinical studies in aging and metabolic disorders	Lack of large randomized controlled trials and long-term outcome data
Direct glutathione supplementation	Increases systemic GSH availability	Oral formulations show inconsistent efficacy; liposomal and intravenous formulations may improve delivery	Limited bioavailability, uncertain tissue penetration, insufficient clinical evidence
Nrf2 activators	Enhance endogenous antioxidant defense and glutathione biosynthesis	Strong experimental evidence; established clinical application in selected disorders (e.g., dimethyl fumarate in multiple sclerosis)	Therapeutic efficacy varies among diseases; long-term safety and disease-specific benefits require further study
Dietary interventions	Supply sulfur-containing amino acids and antioxidant nutrients supporting GSH metabolism	Epidemiological and experimental evidence supports a beneficial role	Difficult to standardize; influenced by dietary adherence and individual variability
Physical activity	Stimulates endogenous antioxidant adaptation and mitochondrial function	Regular moderate exercise consistently improves redox homeostasis	Effects depend on exercise intensity, duration, and patient characteristics
Emerging approaches (nanocarriers, mitochondria-targeted antioxidants, gene-based strategies)	Improve GSH delivery or selectively modulate intracellular redox pathways	Encouraging preclinical findings	Translation into clinical practice remains at an early stage

Abbreviations: GSH, reduced glutathione; GlyNAC, glycine plus N-acetylcysteine; NAC, N-acetylcysteine; Nrf2, nuclear factor erythroid 2-related factor 2; the table summarizes the evidence discussed in the corresponding sections of the manuscript and is based on the references cited therein.

**Table 6:** Future Research Directions in Glutathione Biology.

Research Area	Current Challenge	Future Direction/Potential Impact
Dynamic GSH assessment	Static biomarkers provide limited information	Longitudinal monitoring using redox metabolomics and magnetic resonance spectroscopy
Mitochondrial glutathione	Limited understanding of mGSH regulation	Development of mitochondria-targeted therapeutic strategies
Epigenetic regulation	Incomplete knowledge of redox gene regulation	Identification of novel therapeutic targets and aging mechanisms
Precision redox medicine	High interindividual variability	Patient stratification based on redox phenotype and biomarkers
Multi-omics and systems biology	Fragmented molecular data	Integrated network analysis for biomarker discovery and precision medicine
Translational clinical research	Limited high-quality clinical evidence	Randomized clinical trials evaluating GSH-targeted interventions

Abbreviations: GSH, glutathione; mGSH, mitochondrial glutathione.

## Data Availability

No new data were created or analyzed in this study. Data sharing is not applicable to this article.

## Abbreviations

The following abbreviations are used in this manuscript:

4-HNE	4-hydroxynonenal
AD	Alzheimer’s disease
Aβ	amyloid-β
APP	amyloid precursor protein
ARE	antioxidant response element
ATP7B	ATPase copper transporting beta
CAT	catalase
CNS	central nervous system
CSE	cystathionine γ-lyase
DMF	dimethyl fumarate
eNOS	endothelial nitric oxide synthase
FFAs	free fatty acids
GCL	glutamate–cysteine ligase
GCLC	catalytic subunit of GCL
GCLM	modulatory subunit of GCL
GGT	γ-glutamyl transpeptidase
GlyNAC	glycine and N-acetylcysteine
GPx	glutathione peroxidase
*GPX*	glutathione peroxidase gene
GR	glutathione reductase
GS	glutathione synthetase
GSH	Reduced glutathione
*GSR*	glutathione-disulfide reductase gene
GSSG	glutathione disulfide (oxidized form)
GST	glutathione S-transferase
HD	Huntington’s disease
IL-1β	interleukin-1 beta
IRS	insulin receptor substrate
JNK	c-Jun N-terminal kinase
Keap1	Kelch-like ECH-associated protein 1
MAPK	mitogen-activated protein kinase
MASH	metabolic dysfunction-associated steatohepatitis
MASLD	metabolic dysfunction-associated steatotic liver disease
MDA	malondialdehyde
mGSH	mitochondrial glutathione
mHTT	mutant huntingtin protein
MRS	magnetic resonance spectroscopy
MS	multiple sclerosis
NAC	N-acetylcysteine
NADPH	nicotinamide adenine dinucleotide phosphate
NAFLD	nonalcoholic fatty liver disease
NASH	nonalcoholic steatohepatitis
NF-κB	nuclear factor kappa-light-chain-enhancer of activated B cells
NO	nitric oxide
Nrf2	nuclear factor erythroid 2-related factor 2
PD	Parkinson’s disease
PGC-1α	Peroxisome proliferator-activated receptor gamma coactivator 1-alpha
RNS	reactive nitrogen species
ROS	reactive oxygen species
SOD2	superoxide dismutase 2
SNpc	substantia nigra pars compacta
T2D	Type 2 diabetes
TNF-α	tumor necrosis factor-alpha
WD	Wilson’s disease

## References

[1] Forman H.J., Zhang H. (2021). Targeting Oxidative Stress in Disease: Promise and Limitations of Antioxidant Therapy. Nat. Rev. Drug Discov..

[2] Lapenna D. (2023). Glutathione and Glutathione-Dependent Enzymes: From Biochemistry to Gerontology and Successful Aging. Ageing Res. Rev..

[3] Mas-Bargues C., García-Domínguez E., Borrás C. (2022). Recent Approaches to Determine Static and Dynamic Redox State-Related Parameters. Antioxidants.

[4] Vašková J., Kočan L., Vaško L., Perjési P. (2023). Glutathione-Related Enzymes and Proteins: A Review. Molecules.

[5] Trigo D., Avelar C., Fernandes M., Sá J., da Cruz e Silva O. (2022). Mitochondria, Energy, and Metabolism in Neuronal Health and Disease. FEBS Lett..

[6] Harrington J.S., Ryter S.W., Plataki M., Price D.R., Choi A.M.K. (2023). Mitochondria in Health, Disease, and Aging. Physiol. Rev..

[7] Picca A., Ferri E., Calvani R., Coelho-Júnior H.J., Marzetti E., Arosio B. (2022). Age-Associated Glia Remodeling and Mitochondrial Dysfunction in Neurodegeneration: Antioxidant Supplementation as a Possible Intervention. Nutrients.

[8] Zhang W., Xiao D., Mao Q., Xia H. (2023). Role of Neuroinflammation in Neurodegeneration Development. Signal Transduct. Target. Ther..

[9] Lin M., Liu N., Qin Z., Wang Y. (2022). Mitochondrial-Derived Damage-Associated Molecular Patterns Amplify Neuroinflammation in Neurodegenerative Diseases. Acta Pharmacol. Sin..

[10] Knudsen E., Tadje J., Coggins C., Venketaraman V. (2026). Glutathione and Neurodegenerative Diseases: Immunopharmacological Implications. Front. Pharmacol..

[11] Giustarini D., Milzani A., Dalle-Donne I., Rossi R. (2023). How to Increase Cellular Glutathione. Antioxidants.

[12] Hristov B.D. (2022). The Role of Glutathione Metabolism in Chronic Illness Development and Its Potential Use as a Novel Therapeutic Target. Cureus.

[13] Labarrere C.A., Kassab G.S. (2022). Glutathione: A Samsonian Life-Sustaining Small Molecule That Protects against Oxidative Stress, Ageing and Damaging Inflammation. Front. Nutr..

[14] Shukla D., Goel A., Mandal P.K., Joon S., Punjabi K., Arora Y., Kumar R., Mehta V.S., Singh P., Maroon J.C. (2023). Glutathione Depletion and Concomitant Elevation of Susceptibility in Patients with Parkinson’s Disease: State-of-the-Art MR Spectroscopy and Neuropsychological Study. ACS Chem. Neurosci..

[15] Vrettou S., Wirth B. (2022). S-Glutathionylation and S-Nitrosylation in Mitochondria: Focus on Homeostasis and Neurodegenerative Diseases. Int. J. Mol. Sci..

[16] Ansari M.A., Scheff S.W. (2010). Oxidative Stress in the Progression of Alzheimer Disease in the Frontal Cortex. J. Neuropathol. Exp. Neurol..

[17] Butterfield D.A., Halliwell B. (2019). Oxidative Stress, Dysfunctional Glucose Metabolism and Alzheimer Disease. Nat. Rev. Neurosci..

[18] Gu J., Wu Y., Huang W., Fan X., Chen X., Zhou B., Lin Z., Feng X. (2022). Effect of Vitamin D on Oxidative Stress and Serum Inflammatory Factors in the Patients with Type 2 Diabetes. Clin. Lab. Anal..

[19] Banik S., Ghosh A. (2021). The Association of Oxidative Stress Biomarkers with Type 2 Diabetes Mellitus: A Systematic Review and Meta-analysis. Health Sci. Rep..

[20] Dawi J., Misakyan Y., Affa S., Kades S., Narasimhan A., Hajjar F., Besser M., Tumanyan K., Venketaraman V. (2024). Oxidative Stress, Glutathione Insufficiency, and Inflammatory Pathways in Type 2 Diabetes Mellitus: Implications for Therapeutic Interventions. Biomedicines.

[21] Sies H., Jones D.P. (2020). Reactive Oxygen Species (ROS) as Pleiotropic Physiological Signalling Agents. Nat. Rev. Mol. Cell Biol..

[22] Chen X., Zhou Q., Chen H., Bai J., An R., Zhang K., Zhang X., An H., Zhang J., Wang Y. (2024). Glutathione Induces Keap1 S-Glutathionylation and Mitigates Oscillating Glucose-Induced β-Cell Dysfunction by Activating Nrf2. Antioxidants.

[23] Liu Y., Liu S., Tomar A., Yen F.S., Unlu G., Ropek N., Weber R.A., Wang Y., Khan A., Gad M. (2023). Autoregulatory Control of Mitochondrial Glutathione Homeostasis. Science.

[24] Chen T.-H., Wang H.-C., Chang C.-J., Lee S.-Y. (2024). Mitochondrial Glutathione in Cellular Redox Homeostasis and Disease Manifestation. Int. J. Mol. Sci..

[25] Kobayashi E.H., Suzuki T., Funayama R., Nagashima T., Hayashi M., Sekine H., Tanaka N., Moriguchi T., Motohashi H., Nakayama K. (2016). Nrf2 Suppresses Macrophage Inflammatory Response by Blocking Proinflammatory Cytokine Transcription. Nat. Commun..

[26] Tkaczenko H., Kurhaluk N. (2025). Antioxidant-Rich Functional Foods and Exercise: Unlocking Metabolic Health Through Nrf2 and Related Pathways. Int. J. Mol. Sci..

[27] Minich D.M., Brown B.I. (2019). A Review of Dietary (Phyto)Nutrients for Glutathione Support. Nutrients.

[28] Radak Z., Zhao Z., Koltai E., Ohno H., Atalay M. (2013). Oxygen Consumption and Usage During Physical Exercise: The Balance Between Oxidative Stress and ROS-Dependent Adaptive Signaling. Antioxid. Redox Signal..

[29] Anderson M.E. (2022). Assay of the Enzymes of Glutathione Biosynthesis. Anal. Biochem..

[30] Zhao L., Xia W., Jiang P. (2021). CREB1 and ATF1 Negatively Regulate Glutathione Biosynthesis Sensitizing Cells to Oxidative Stress. Front. Cell Dev. Biol..

[31] Chen Y., Shertzer H.G., Schneider S.N., Nebert D.W., Dalton T.P. (2005). Glutamate Cysteine Ligase Catalysis. J. Biol. Chem..

[32] McCann M.S., Maguire-Zeiss K.A. (2021). Environmental Toxicants in the Brain: A Review of Astrocytic Metabolic Dysfunction. Environ. Toxicol. Pharmacol..

[33] Chacón E., Grünwaldt G., Marmisolle I., Martínez J., Quijano C. (2026). Redox Metabolism in Cell Senescence: Focusing on Contributions from the Metabolomic Field. Front. Mol. Biosci..

[34] Tretter V., Hochreiter B., Zach M.L., Krenn K., Klein K.U. (2021). Understanding Cellular Redox Homeostasis: A Challenge for Precision Medicine. Int. J. Mol. Sci..

[35] Kalinina E. (2024). Glutathione-Dependent Pathways in Cancer Cells. Int. J. Mol. Sci..

[36] Asantewaa G., Tuttle E.T., Ward N.P., Kang Y.P., Kim Y., Kavanagh M.E., Girnius N., Chen Y., Rodriguez K., Hecht F. (2024). Glutathione Synthesis in the Mouse Liver Supports Lipid Abundance through NRF2 Repression. Nat. Commun..

[37] Liu X., Ma Q., Jia Z., Zhou Y., Zou C., Xiao Y., Chen Y., Ma C., Song L., Yang J. (2025). ISG15 Enhances the Activity of γ-Glutamate Cysteine Ligase to Suppress Apoptosis in High Fat Diet-Promoted Hepatocellular Carcinoma. Adv. Sci..

[38] Lu S.C. (2013). Glutathione Synthesis. Biochim. Biophys. Acta (BBA)—Gen. Subj..

[39] Ikeda Y., Fujii J. (2023). The Emerging Roles of γ-Glutamyl Peptides Produced by γ-Glutamyltransferase and the Glutathione Synthesis System. Cells.

[40] Cassier-Chauvat C., Marceau F., Farci S., Ouchane S., Chauvat F. (2023). The Glutathione System: A Journey from Cyanobacteria to Higher Eukaryotes. Antioxidants.

[41] Bonetti L., Horkova V., Grusdat M., Longworth J., Guerra L., Kurniawan H., Franchina D.G., Soriano-Baguet L., Binsfeld C., Verschueren C. (2024). A Th17 Cell-Intrinsic Glutathione/Mitochondrial-IL-22 Axis Protects against Intestinal Inflammation. Cell Metab..

[42] Zhu H., Cheng Y., Wang X., Yang X., Liu M., Liu J., Liu S., Wang H., Zhang A., Li R. (2023). Gss Deficiency Causes Age-Related Fertility Impairment via ROS-Triggered Ferroptosis in the Testes of Mice. Cell Death Dis..

[43] Couto N., Wood J., Barber J. (2016). The Role of Glutathione Reductase and Related Enzymes on Cellular Redox Homoeostasis Network. Free Radic. Biol. Med..

[44] Vairetti M., Di Pasqua L.G., Cagna M., Richelmi P., Ferrigno A., Berardo C. (2021). Changes in Glutathione Content in Liver Diseases: An Update. Antioxidants.

[45] Itoh K., Chiba T., Takahashi S., Ishii T., Igarashi K., Katoh Y., Oyake T., Hayashi N., Satoh K., Hatayama I. (1997). An Nrf2/Small Maf Heterodimer Mediates the Induction of Phase II Detoxifying Enzyme Genes through Antioxidant Response Elements. Biochem. Biophys. Res. Commun..

[46] Ngo V., Karunatilleke N.C., Brickenden A., Choy W.-Y., Duennwald M.L. (2022). Oxidative Stress-Induced Misfolding and Inclusion Formation of Nrf2 and Keap1. Antioxidants.

[47] Wijaya L.S., Rau C., Braun T.S., Marangoz S., Spegg V., Vlasveld M., Albrecht W., Brecklinghaus T., Kamp H., Beltman J.B. (2022). Stimulation of de Novo Glutathione Synthesis by Nitrofurantoin for Enhanced Resilience of Hepatocytes. Cell Biol. Toxicol..

[48] Wufuer R., Fan Z., Liu K., Zhang Y. (2021). Differential Yet Integral Contributions of Nrf1 and Nrf2 in the Human HepG2 Cells on Antioxidant Cytoprotective Response against Tert-Butylhydroquinone as a Pro-Oxidative Stressor. Antioxidants.

[49] Schnell M.R., Zhai T., Ragwan E.R., Jung H., Zhang J., Lagalante A.F., Kung Y., Kraut D.A., Huang Z., Eggler A.L. (2025). KEAP1 C151 Active Site Catalysis Drives Electrophilic Signaling to Upregulate Cytoprotective Enzyme Expression. Redox Biol..

[50] Morgenstern C., Lastres-Becker I., Demirdöğen B.C., Costa V.M., Daiber A., Foresti R., Motterlini R., Kalyoncu S., Arioz B.I., Genc S. (2024). Biomarkers of NRF2 Signalling: Current Status and Future Challenges. Redox Biol..

[51] Wang R., Liang L., Matsumoto M., Iwata K., Umemura A., He F. (2023). Reactive Oxygen Species and NRF2 Signaling, Friends or Foes in Cancer?. Biomolecules.

[52] Murakami S., Kusano Y., Okazaki K., Akaike T., Motohashi H. (2026). NRF2 Signalling in Cytoprotection and Metabolism. Br. J. Pharmacol..

[53] Federici L., Masulli M., De Laurenzi V., Allocati N. (2024). The Role of S-Glutathionylation in Health and Disease: A Bird’s Eye View. Nutrients.

[54] Mustafa Rizvi S.H., Shao D., Tsukahara Y., Pimentel D.R., Weisbrod R.M., Hamburg N.M., McComb M.E., Matsui R., Bachschmid M.M. (2021). Oxidized GAPDH Transfers S-Glutathionylation to a Nuclear Protein Sirtuin-1 Leading to Apoptosis. Free Radic. Biol. Med..

[55] Guo Y., Liu Y., Zhao S., Xu W., Li Y., Zhao P., Wang D., Cheng H., Ke Y., Zhang X. (2021). Oxidative Stress-Induced FABP5 S-Glutathionylation Protects against Acute Lung Injury by Suppressing Inflammation in Macrophages. Nat. Commun..

[56] El Kodsi D.N., Tokarew J.M., Sengupta R., Lengacher N.A., Chatterji A., Nguyen A.P., Boston H., Jiang Q., Palmberg C., Pileggi C. (2023). Parkin Coregulates Glutathione Metabolism in Adult Mammalian Brain. Acta Neuropathol. Commun..

[57] Sun X., Guo C., Huang C., Lv N., Chen H., Huang H., Zhao Y., Sun S., Zhao D., Tian J. (2024). GSTP Alleviates Acute Lung Injury by S-Glutathionylation of KEAP1 and Subsequent Activation of NRF2 Pathway. Redox Biol..

[58] Vázquez-Meza H., Vilchis-Landeros M.M., Vázquez-Carrada M., Uribe-Ramírez D., Matuz-Mares D. (2023). Cellular Compartmentalization, Glutathione Transport and Its Relevance in Some Pathologies. Antioxidants.

[59] Lin H., Wang L., Jiang X., Wang J. (2024). Glutathione Dynamics in Subcellular Compartments and Implications for Drug Development. Curr. Opin. Chem. Biol..

[60] Scirè A., Cianfruglia L., Minnelli C., Bartolini D., Torquato P., Principato G., Galli F., Armeni T. (2019). Glutathione Compartmentalization and Its Role in Glutathionylation and Other Regulatory Processes of Cellular Pathways. BioFactors.

[61] Ribas V., García-Ruiz C., Fernández-Checa J.C. (2014). Glutathione and Mitochondria. Front. Pharmacol..

[62] Wang Y., Yen F.S., Zhu X.G., Timson R.C., Weber R., Xing C., Liu Y., Allwein B., Luo H., Yeh H.-W. (2021). SLC25A39 Is Necessary for Mitochondrial Glutathione Import in Mammalian Cells. Nature.

[63] Shi X., Reinstadler B., Shah H., To T.-L., Byrne K., Summer L., Calvo S.E., Goldberger O., Doench J.G., Mootha V.K. (2022). Combinatorial GxGxE CRISPR Screen Identifies SLC25A39 in Mitochondrial Glutathione Transport Linking Iron Homeostasis to OXPHOS. Nat. Commun..

[64] Marí M., Morales A., Colell A., García-Ruiz C., Kaplowitz N., Fernández-Checa J.C. (2013). Mitochondrial Glutathione: Features, Regulation and Role in Disease. Biochim. Biophys. Acta (BBA)—Gen. Subj..

[65] Nagana Gowda G.A., Abell L., Tian R., Raftery D. (2023). Whole Body Distribution of Labile Coenzymes and Antioxidants in a Mouse Model as Visualized Using^1^ H NMR Spectroscopy. Anal. Chem..

[66] Georgiou-Siafis S.K., Tsiftsoglou A.S. (2023). The Key Role of GSH in Keeping the Redox Balance in Mammalian Cells: Mechanisms and Significance of GSH in Detoxification via Formation of Conjugates. Antioxidants.

[67] Pérez-Sala D., Pajares M.A. (2023). Appraising the Role of Astrocytes as Suppliers of Neuronal Glutathione Precursors. Int. J. Mol. Sci..

[68] Jomova K., Raptova R., Alomar S.Y., Alwasel S.H., Nepovimova E., Kuca K., Valko M. (2023). Reactive Oxygen Species, Toxicity, Oxidative Stress, and Antioxidants: Chronic Diseases and Aging. Arch. Toxicol..

[69] Guan X. (2024). Glutathione Transporter as a Target for Brain Drug Delivery. Med. Chem. Res..

[70] Schichlein K.D., Smith G.J., Jaspers I. (2023). Protective Effects of Inhaled Antioxidants against Air Pollution-Induced Pathological Responses. Respir. Res..

[71] Gopika M.G., Gopidas S., Jayan G.S., Arathy P.S., Saraswathyamma B. (2024). Unveiling Thiol Biomarkers: Glutathione and Cysteamine. Clin. Chim. Acta.

[72] Marrone J., Danielli M., Gaspari C.I., Capiglioni A.M., Marinelli R.A. (2021). Aquaporin Gene Transfer for Hepatocellular Cholestasis. Biochimie.

[73] Jiang X., Zhou Q., Du B., Li S., Huang Y., Chi Z., Lee W.M., Yu M., Zheng J. (2021). Noninvasive Monitoring of Hepatic Glutathione Depletion through Fluorescence Imaging and Blood Testing. Sci. Adv..

[74] Sotgia S., Fois A.G., Paliogiannis P., Carru C., Mangoni A.A., Zinellu A. (2021). Methodological Fallacies in the Determination of Serum/Plasma Glutathione Limit Its Translational Potential in Chronic Obstructive Pulmonary Disease. Molecules.

[75] Chia S.B., Elko E.A., Aboushousha R., Manuel A.M., Van De Wetering C., Druso J.E., Van Der Velden J., Seward D.J., Anathy V., Irvin C.G. (2020). Dysregulation of the Glutaredoxin/ *S*- Glutathionylation Redox Axis in Lung Diseases. Am. J. Physiol. -Cell Physiol..

[76] Hall A.M., Trepiccione F., Unwin R.J. (2022). Drug Toxicity in the Proximal Tubule: New Models, Methods and Mechanisms. Pediatr. Nephrol..

[77] Lash L.H. (2024). Renal Glutathione: Dual Roles as Antioxidant Protector and Bioactivation Promoter. Biochem. Pharmacol..

[78] Dringen R., Arend C. (2025). Glutathione Metabolism of the Brain—The Role of Astrocytes. J. Neurochem..

[79] Mulica P., Grünewald A., Pereira S.L. (2021). Astrocyte-Neuron Metabolic Crosstalk in Neurodegeneration: A Mitochondrial Perspective. Front. Endocrinol..

[80] Aoyama K. (2021). Glutathione in the Brain. Int. J. Mol. Sci..

[81] Lizák B., Birk J., Zana M., Kosztyi G., Kratschmar D.V., Odermatt A., Zimmermann R., Geiszt M., Appenzeller-Herzog C., Bánhegyi G. (2020). Ca2+ Mobilization-Dependent Reduction of the Endoplasmic Reticulum Lumen Is Due to Influx of Cytosolic Glutathione. BMC Biol..

[82] Costa C.F., Lismont C., Chornyi S., Koster J., Li H., Hussein M.A.F., Van Veldhoven P.P., Waterham H.R., Fransen M. (2024). The Solute Carrier SLC25A17 Sustains Peroxisomal Redox Homeostasis in Diverse Mammalian Cell Lines. Free Radic. Biol. Med..

[83] Marí M., De Gregorio E., De Dios C., Roca-Agujetas V., Cucarull B., Tutusaus A., Morales A., Colell A. (2020). Mitochondrial Glutathione: Recent Insights and Role in Disease. Antioxidants.

[84] Bonora M., Giorgi C., Pinton P. (2022). Molecular Mechanisms and Consequences of Mitochondrial Permeability Transition. Nat. Rev. Mol. Cell Biol..

[85] Pitton Rissardo J., McGarry A., Shi Y., Fornari Caprara A.L., Kannarkat G.T. (2025). Alpha-Synuclein Neurobiology in Parkinson’s Disease: A Comprehensive Review of Its Role, Mechanisms, and Therapeutic Perspectives. Brain Sci..

[86] Coukos R., Krainc D. (2024). Key Genes and Convergent Pathogenic Mechanisms in Parkinson Disease. Nat. Rev. Neurosci..

[87] Morris H.R., Spillantini M.G., Sue C.M., Williams-Gray C.H. (2024). The Pathogenesis of Parkinson’s Disease. Lancet.

[88] Dexter D.T., Jenner P. (2013). Parkinson Disease: From Pathology to Molecular Disease Mechanisms. Free Radic. Biol. Med..

[89] Dias V., Junn E., Mouradian M.M. (2013). The Role of Oxidative Stress in Parkinson’s Disease. J. Park. Dis..

[90] Moon H.E., Paek S.H. (2015). Mitochondrial Dysfunction in Parkinson’s Disease. Exp. Neurobiol..

[91] Sian J., Dexter D.T., Lees A.J., Daniel S., Agid Y., Javoy-Agid F., Jenner P., Marsden C.D. (1994). Alterations in Glutathione Levels in Parkinson’s Disease and Other Neurodegenerative Disorders Affecting Basal Ganglia. Ann. Neurol..

[92] Bjørklund G., Peana M., Maes M., Dadar M., Severin B. (2021). The Glutathione System in Parkinson’s Disease and Its Progression. Neurosci. Biobehav. Rev..

[93] Martinez-Banaclocha M.A. (2023). Targeting the Cysteine Redox Proteome in Parkinson’s Disease: The Role of Glutathione Precursors and Beyond. Antioxidants.

[94] Jenner P., Dexter D.T., Sian J., Schapira A.H.V., Marsden C.D., The Royal Kings And Queens Parkinson’s Disease Research Group (1992). Oxidative Stress as a Cause of Nigral Cell Death in Parkinson’s Disease and Incidental Lewy Body Disease. Ann. Neurol..

[95] Schapira A.H.V., Cooper J.M., Dexter D., Clark J.B., Jenner P., Marsden C.D. (1990). Mitochondrial Complex I Deficiency in Parkinson’s Disease. J. Neurochem..

[96] Ni A., Ernst C. (2022). Evidence That Substantia Nigra Pars Compacta Dopaminergic Neurons Are Selectively Vulnerable to Oxidative Stress Because They Are Highly Metabolically Active. Front. Cell. Neurosci..

[97] Burbulla L.F., Song P., Mazzulli J.R., Zampese E., Wong Y.C., Jeon S., Santos D.P., Blanz J., Obermaier C.D., Strojny C. (2017). Dopamine Oxidation Mediates Mitochondrial and Lysosomal Dysfunction in Parkinson’s Disease. Science.

[98] Schildknecht S., Gerding H.R., Karreman C., Drescher M., Lashuel H.A., Outeiro T.F., Di Monte D.A., Leist M. (2013). Oxidative and Nitrative Alpha-synuclein Modifications and Proteostatic Stress: Implications for Disease Mechanisms and Interventions in Synucleinopathies. J. Neurochem..

[99] Guo J., Zhao X., Li Y., Li G., Liu X. (2018). Damage to Dopaminergic Neurons by Oxidative Stress in Parkinson’s Disease (Review). Int. J. Mol. Med..

[100] Chakrabarti S., Bisaglia M. (2023). Oxidative Stress and Neuroinflammation in Parkinson’s Disease: The Role of Dopamine Oxidation Products. Antioxidants.

[101] Risiglione P., Zinghirino F., Di Rosa M.C., Magrì A., Messina A. (2021). Alpha-Synuclein and Mitochondrial Dysfunction in Parkinson’s Disease: The Emerging Role of VDAC. Biomolecules.

[102] Haseena P.A., Diwakar L., Ravindranath V. (2022). Protein Glutathionylation and Glutaredoxin: Role in Neurodegenerative Diseases. Antioxidants.

[103] Goyal P., Baldaniya L., Tyagi L.K., Joshi K.K., Ballal S., Sabarivani A., Ray S., Nathiya D., Chauhan A.S., Gulati M. (2026). Neuroimmune Interactions in Neurodegeneration: The Role of Microglia in Alzheimer’s and Parkinson’s Disease Pathogenesis. Brain Sci..

[104] Liu E., Zhang Y., Wang J.-Z. (2024). Updates in Alzheimer’s Disease: From Basic Research to Diagnosis and Therapies. Transl. Neurodegener..

[105] Tzioras M., McGeachan R.I., Durrant C.S., Spires-Jones T.L. (2023). Synaptic Degeneration in Alzheimer Disease. Nat. Rev. Neurol..

[106] Hein Z.M., Karikalan B., Gopalakrishna P.K., Dhevi K., Alkatiri A., Hussan F., Mohd Moklas M.A., Jagadeesan S., Che Ramli M.D., Che Mohd Nassir C.M.N. (2026). Toward a Unified Framework in Molecular Neurobiology of Alzheimer’s Disease: Revisiting the Pathophysiological Hypotheses. Mol. Neurobiol..

[107] Bajinka O., Jallow L., Ozdemir G. (2026). A Multi-Target Therapeutic Framework for Alzheimer’s Disease: An Integrative Mechanistic Review. Neuroscience.

[108] Rajendran K., Krishnan U.M. (2024). Mechanistic Insights and Emerging Therapeutic Stratagems for Alzheimer’s Disease. Ageing Res. Rev..

[109] Jurcău M.C., Andronie-Cioara F.L., Jurcău A., Marcu F., Ţiț D.M., Pașcalău N., Nistor-Cseppentö D.C. (2022). The Link between Oxidative Stress, Mitochondrial Dysfunction and Neuroinflammation in the Pathophysiology of Alzheimer’s Disease: Therapeutic Implications and Future Perspectives. Antioxidants.

[110] Kazemeini S., Nadeem-Tariq A., Shih R., Rafanan J., Ghani N., Vida T.A. (2024). From Plaques to Pathways in Alzheimer’s Disease: The Mitochondrial-Neurovascular-Metabolic Hypothesis. Int. J. Mol. Sci..

[111] Chen J.J., Thiyagarajah M., Song J., Chen C., Herrmann N., Gallagher D., Rapoport M.J., Black S.E., Ramirez J., Andreazza A.C. (2022). Altered Central and Blood Glutathione in Alzheimer’s Disease and Mild Cognitive Impairment: A Meta-Analysis. Alzheimers Res. Ther..

[112] Mandal P.K., Saharan S., Tripathi M., Murari G. (2015). Brain Glutathione Levels—A Novel Biomarker for Mild Cognitive Impairment and Alzheimer’s Disease. Biol. Psychiatry.

[113] McKiernan E., Su L., O’Brien J. (2023). MRS in Neurodegenerative Dementias, Prodromal Syndromes and At-risk States: A Systematic Review of the Literature. NMR Biomed..

[114] Shukla D., Mandal P.K., Tripathi M., Vishwakarma G., Mishra R., Sandal K. (2020). Quantitation of in Vivo Brain Glutathione Conformers in Cingulate Cortex among Age-matched Control, MCI, and AD Patients Using MEGA-PRESS. Hum. Brain Mapp..

[115] Karran E., Mercken M., Strooper B.D. (2011). The Amyloid Cascade Hypothesis for Alzheimer’s Disease: An Appraisal for the Development of Therapeutics. Nat. Rev. Drug Discov..

[116] Chi L., Ke Y., Luo C., Gozal D., Liu R. (2007). Depletion of Reduced Glutathione Enhances Motor Neuron Degeneration in Vitro and in Vivo. Neuroscience.

[117] Sabens Liedhegner E.A., Gao X.-H., Mieyal J.J. (2012). Mechanisms of Altered Redox Regulation in Neurodegenerative Diseases—Focus on S-Glutathionylation. Antioxid. Redox Signal..

[118] Barbero-Camps E., Fernández A., Martínez L., Fernández-Checa J.C., Colell A. (2013). APP/PS1 Mice Overexpressing SREBP-2 Exhibit Combined Aβ Accumulation and Tau Pathology Underlying Alzheimer’s Disease. Hum. Mol. Genet..

[119] Dalle-Donne I., Rossi R., Colombo G., Giustarini D., Milzani A. (2009). Protein S-Glutathionylation: A Regulatory Device from Bacteria to Humans. Trends Biochem. Sci..

[120] Ghezzi P. (2013). Protein Glutathionylation in Health and Disease. Biochim. Biophys. Acta (BBA)—Gen. Subj..

[121] Shelton M.D., Chock P.B., Mieyal J.J. (2005). Glutaredoxin: Role in Reversible Protein *S* -Glutathionylation and Regulation of Redox Signal Transduction and Protein Translocation. Antioxid. Redox Signal..

[122] Azmal M., Paul J.K., Prima F.S., Haque A.N.M.S.N.B., Meem M., Ghosh A. (2025). Microglial Dysfunction in Alzheimer’s Disease: Mechanisms, Emerging Therapies, and Future Directions. Exp. Neurol..

[123] Lomelí Martínez S.M., Pacheco Moisés F.P., Bitzer-Quintero O.K., Ramírez-Jirano J., Delgado-Lara D.L.C., Cortés Trujillo I., Torres Jasso J.H., Salazar-Flores J., Torres-Sánchez E.D. (2025). Effect of N-Acetyl Cysteine as an Adjuvant Treatment in Alzheimer’s Disease. Brain Sci..

[124] Johnson J.A., Johnson D.A., Kraft A.D., Calkins M.J., Jakel R.J., Vargas M.R., Chen P. (2008). The Nrf2–ARE Pathway: An Indicator and Modulator of Oxidative Stress in Neurodegeneration. Ann. N. Y. Acad. Sci..

[125] Walker F.O. (2007). Huntington’s Disease. Lancet.

[126] Saso L., Ates I., Tunc R., Yilmaz B., Gallorini M., Carradori S., Suzen S. (2025). Modulation of Nrf2 and Mitochondrial Function: Pharmacological Implications. Pharmaceuticals.

[127] Brandes M.S., Zweig J.A., Tang A., Gray N.E. (2021). NRF2 Activation Ameliorates Oxidative Stress and Improves Mitochondrial Function and Synaptic Plasticity, and in A53T α-Synuclein Hippocampal Neurons. Antioxidants.

[128] Dinkova-Kostova A.T., Kostov R.V., Kazantsev A.G. (2018). The Role of Nrf2 Signaling in Counteracting Neurodegenerative Diseases. FEBS J..

[129] Pocernich C.B., Butterfield D.A. (2012). Elevation of Glutathione as a Therapeutic Strategy in Alzheimer Disease. Biochim. Biophys. Acta (BBA)—Mol. Basis Dis..

[130] Mulè S., Ferrari S., Rosso G., Brovero A., Botta M., Congiusta A., Galla R., Molinari C., Uberti F. (2024). The Combined Antioxidant Effects of N-Acetylcysteine, Vitamin D3, and Glutathione from the Intestinal–Neuronal In Vitro Model. Foods.

[131] Persson T., Popescu B.O., Cedazo-Minguez A. (2014). Oxidative Stress in Alzheimer’s Disease: Why Did Antioxidant Therapy Fail?. Oxidative Med. Cell. Longev..

[132] Gonzalez Rojas N., Cesarini M.E., Peker G., Da Prat G.A., Etcheverry J.L., Gatto E.M. (2022). Review of Huntington’s Disease: From Basics to Advances in Diagnosis and Treatment. J. Neurol. Res..

[133] Alqahtani T., Deore S.L., Kide A.A., Shende B.A., Sharma R., Dadarao Chakole R., Nemade L.S., Kishor Kale N., Borah S., Shrikant Deokar S. (2023). Mitochondrial Dysfunction and Oxidative Stress in Alzheimer’s Disease, and Parkinson’s Disease, Huntington’s Disease and Amyotrophic Lateral Sclerosis -An Updated Review. Mitochondrion.

[134] Jiménez-Jiménez F.J., Alonso-Navarro H., García-Martín E., Cárcamo-Fonfría A., Caballero-Muñoz M.D.M., Agúndez J.A.G. (2025). Oxidative Stress in Huntington’s Disease. Biomolecules.

[135] Zheng J., Winderickx J., Franssens V., Liu B. (2018). A Mitochondria-Associated Oxidative Stress Perspective on Huntington’s Disease. Front. Mol. Neurosci..

[136] Cui L., Jeong H., Borovecki F., Parkhurst C.N., Tanese N., Krainc D. (2006). Transcriptional Repression of PGC-1α by Mutant Huntingtin Leads to Mitochondrial Dysfunction and Neurodegeneration. Cell.

[137] Tsunemi T., Ashe T.D., Morrison B.E., Soriano K.R., Au J., Roque R.A.V., Lazarowski E.R., Damian V.A., Masliah E., La Spada A.R. (2012). PGC-1α Rescues Huntington’s Disease Proteotoxicity by Preventing Oxidative Stress and Promoting TFEB Function. Sci. Transl. Med..

[138] St-Pierre J., Drori S., Uldry M., Silvaggi J.M., Rhee J., Jäger S., Handschin C., Zheng K., Lin J., Yang W. (2006). Suppression of Reactive Oxygen Species and Neurodegeneration by the PGC-1 Transcriptional Coactivators. Cell.

[139] Lu S.C. (2009). Regulation of Glutathione Synthesis. Mol. Asp. Med..

[140] Paul B.D., Sbodio J.I., Xu R., Vandiver M.S., Cha J.Y., Snowman A.M., Snyder S.H. (2014). Cystathionine γ-Lyase Deficiency Mediates Neurodegeneration in Huntington’s Disease. Nature.

[141] Paul B.D., Sbodio J.I., Snyder S.H. (2022). Mutant Huntingtin Derails Cysteine Metabolism in Huntington’s Disease at Both Transcriptional and Post-Translational Levels. Antioxidants.

[142] Ribeiro M., Rosenstock T.R., Cunha-Oliveira T., Ferreira I.L., Oliveira C.R., Rego A.C. (2012). Glutathione Redox Cycle Dysregulation in Huntington’s Disease Knock-in Striatal Cells. Free Radic. Biol. Med..

[143] Browne S.E., Ferrante R.J., Beal M.F. (1999). Oxidative Stress in Huntington’s Disease. Brain Pathol..

[144] Túnez I., Tasset I., Pérez-De La Cruz V., Santamaría A. (2010). 3-Nitropropionic Acid as a Tool to Study the Mechanisms Involved in Huntington’s Disease: Past, Present and Future. Molecules.

[145] Klepac N., Relja M., Klepac R., Hećimović S., Babić T., Trkulja V. (2007). Oxidative Stress Parameters in Plasma of Huntington’s Disease Patients, Asymptomatic Huntington’s Disease Gene Carriers and Healthy Subjects: A Cross-Sectional Study. J. Neurol..

[146] Palpagama T., Mills A.R., Ferguson M.W., Vikas Ankeal P., Turner C., Tippett L., Van Der Werf B., Waldvogel H.J., Faull R.L.M., Kwakowsky A. (2023). Microglial and Astrocytic Responses in the Human Midcingulate Cortex in Huntington’s Disease. Ann. Neurol..

[147] O’Regan G.C., Farag S.H., Casey C.S., Wood-Kaczmar A., Pocock J.M., Tabrizi S.J., Andre R. (2021). Human Huntington’s Disease Pluripotent Stem Cell-Derived Microglia Develop Normally but Are Abnormally Hyper-Reactive and Release Elevated Levels of Reactive Oxygen Species. J. Neuroinflammation.

[148] Palpagama T.H., Waldvogel H.J., Faull R.L.M., Kwakowsky A. (2019). The Role of Microglia and Astrocytes in Huntington’s Disease. Front. Mol. Neurosci..

[149] Sapp E., Kegel K.B., Aronin N., Hashikawa T., Uchiyama Y., Tohyama K., Bhide P.G., Vonsattel J.P., Difiglia M. (2001). Early and Progressive Accumulation of Reactive Microglia in the Huntington Disease Brain. J. Neuropathol. Exp. Neurol..

[150] Tucci P., Lattanzi R., Severini C., Saso L. (2022). Nrf2 Pathway in Huntington’s Disease (HD): What Is Its Role?. Int. J. Mol. Sci..

[151] Calkins M.J., Johnson D.A., Townsend J.A., Vargas M.R., Dowell J.A., Williamson T.P., Kraft A.D., Lee J.-M., Li J., Johnson J.A. (2009). The Nrf2/ARE Pathway as a Potential Therapeutic Target in Neurodegenerative Disease. Antioxid. Redox Signal..

[152] Ellrichmann G., Petrasch-Parwez E., Lee D.-H., Reick C., Arning L., Saft C., Gold R., Linker R.A. (2011). Efficacy of Fumaric Acid Esters in the R6/2 and YAC128 Models of Huntington’s Disease. PLoS ONE.

[153] Xun Z., Rivera-Sánchez S., Ayala-Peña S., Lim J., Budworth H., Skoda E.M., Robbins P.D., Niedernhofer L.J., Wipf P., McMurray C.T. (2012). Targeting of XJB-5-131 to Mitochondria Suppresses Oxidative DNA Damage and Motor Decline in a Mouse Model of Huntington’s Disease. Cell Rep..

[154] Wright D.J., Renoir T., Smith Z.M., Frazier A.E., Francis P.S., Thorburn D.R., McGee S.L., Hannan A.J., Gray L.J. (2015). N-Acetylcysteine Improves Mitochondrial Function and Ameliorates Behavioral Deficits in the R6/1 Mouse Model of Huntington’s Disease. Transl. Psychiatry.

[155] Farag M., Tabrizi S.J., Wild E.J. (2025). Huntington’s Disease Clinical Trials Update: March 2025. J. Huntington’s Dis..

[156] Compston A., Coles A. (2008). Multiple Sclerosis. Lancet.

[157] Lassmann H. (2019). Pathogenic Mechanisms Associated With Different Clinical Courses of Multiple Sclerosis. Front. Immunol..

[158] Mahad D.H., Trapp B.D., Lassmann H. (2015). Pathological Mechanisms in Progressive Multiple Sclerosis. Lancet Neurol..

[159] Haider L. (2015). Inflammation, Iron, Energy Failure, and Oxidative Stress in the Pathogenesis of Multiple Sclerosis. Oxidative Med. Cell. Longev..

[160] Van Horssen J., Witte M.E., Schreibelt G., De Vries H.E. (2011). Radical Changes in Multiple Sclerosis Pathogenesis. Biochim. Biophys. Acta (BBA)—Mol. Basis Dis..

[161] Mak T.W., Grusdat M., Duncan G.S., Dostert C., Nonnenmacher Y., Cox M., Binsfeld C., Hao Z., Brüstle A., Itsumi M. (2017). Glutathione Primes T Cell Metabolism for Inflammation. Immunity.

[162] Carvalho A.N., Lim J.L., Nijland P.G., Witte M.E., Van Horssen J. (2014). Glutathione in Multiple Sclerosis: More than Just an Antioxidant?. Mult. Scler. J..

[163] Zhang S.-Y., Gui L.-N., Liu Y.-Y., Shi S., Cheng Y. (2020). Oxidative Stress Marker Aberrations in Multiple Sclerosis: A Meta-Analysis Study. Front. Neurosci..

[164] Choi I.-Y., Lee P., Adany P., Hughes A.J., Belliston S., Denney D.R., Lynch S.G. (2018). In Vivo Evidence of Oxidative Stress in Brains of Patients with Progressive Multiple Sclerosis. Mult. Scler. J..

[165] Hu C., Nydes M., Shanley K.L., Morales Pantoja I.E., Howard T.A., Bizzozero O.A. (2019). Reduced Expression of the Ferroptosis Inhibitor Glutathione Peroxidase-4 in Multiple Sclerosis and Experimental Autoimmune Encephalomyelitis. J. Neurochem..

[166] Bradl M., Lassmann H. (2010). Oligodendrocytes: Biology and Pathology. Acta Neuropathol..

[167] Ying W. (2006). NAD+ and NADH in Cellular Functions and Cell Death. Front. Biosci..

[168] Tonelli C., Chio I.I.C., Tuveson D.A. (2018). Transcriptional Regulation by Nrf2. Antioxid. Redox Signal..

[169] De Haan J.B. (2011). Nrf2 Activators as Attractive Therapeutics for Diabetic Nephropathy. Diabetes.

[170] Racke M., Nicholas J., Boster A., Imitola J., O’Connell C. (2014). Design of Oral Agents for the Management of Multiple Sclerosis: Benefit and Risk Assessment for Dimethyl Fumarate. DDDT Drug Des. Dev. Ther..

[171] Rosito M., Testi C., Parisi G., Cortese B., Baiocco P., Di Angelantonio S. (2020). Exploring the Use of Dimethyl Fumarate as Microglia Modulator for Neurodegenerative Diseases Treatment. Antioxidants.

[172] Monti D.A., Zabrecky G., Leist T.P., Wintering N., Bazzan A.J., Zhan T., Newberg A.B. (2020). N-Acetyl Cysteine Administration Is Associated With Increased Cerebral Glucose Metabolism in Patients With Multiple Sclerosis: An Exploratory Study. Front. Neurol..

[173] Jiménez-Jiménez F.J., Alonso-Navarro H., Salgado-Cámara P., García-Martín E., Agúndez J.A.G. (2024). Antioxidant Therapies in the Treatment of Multiple Sclerosis. Biomolecules.

[174] Krysko K.M., Bischof A., Nourbakhsh B., Henry R.G., Revirajan N., Manguinao M., Nguyen K., Akula A., Li Y., Waubant E. (2021). A Pilot Study of Oxidative Pathways in MS Fatigue: Randomized Trial of N-acetyl Cysteine. Ann. Clin. Transl. Neurol..

[175] Mîndreanu R., Chiș I.C., Sevastre-Berghian A., Login C., Stan A., Stan T., Clichici S., Suciu Ș. (2026). N-Acetylcysteine in Neurological Disorders: A Systematic Review of Clinical and Translational Evidence Across Seven Disorders. Int. J. Mol. Sci..

[176] Członkowska A., Litwin T., Dusek P., Ferenci P., Lutsenko S., Medici V., Rybakowski J.K., Weiss K.H., Schilsky M.L. (2018). Wilson Disease. Nat. Rev. Dis. Prim..

[177] Lorincz M.T. (2018). Wilson Disease and Related Copper Disorders. Handbook of Clinical Neurology.

[178] Gromadzka G., Karpińska A., Przybyłkowski A., Litwin T., Wierzchowska-Ciok A., Dzieżyc K., Chabik G., Członkowska A. (2014). Treatment with D-Penicillamine or Zinc Sulphate Affects Copper Metabolism and Improves but Not Normalizes Antioxidant Capacity Parameters in Wilson Disease. Biometals.

[179] Gromadzka G., Bendykowska M., Przybyłkowski A. (2023). Wilson’s Disease—Genetic Puzzles with Diagnostic Implications. Diagnostics.

[180] Medici V., Karl-Heinz W. (2017). Genetic and Environmental Modifiers of Wilson Disease. Handbook of Clinical Neurology.

[181] Wu F., Wang J., Pu C., Qiao L., Jiang C. (2015). Wilson’s Disease: A Comprehensive Review of the Molecular Mechanisms. Int. J. Mol. Sci..

[182] Zischka H., Lichtmannegger J. (2019). Cellular Copper Toxicity: A Critical Appraisal of Fenton-Chemistry-Based Oxidative Stress in Wilson Disease. Wilson Disease.

[183] Nagasaka H., Inoue I., Inui A., Komatsu H., Sogo T., Murayama K., Murakami T., Yorifuji T., Asayama K., Katayama S. (2006). Relationship Between Oxidative Stress and Antioxidant Systems in the Liver of Patients With Wilson Disease: Hepatic Manifestation in Wilson Disease as a Consequence of Augmented Oxidative Stress. Pediatr. Res..

[184] Gromadzka G., Karpińska A., Szafrański T.K., Litwin T. (2025). Oxidative Stress and Psychiatric Symptoms in Wilson’s Disease. Int. J. Mol. Sci..

[185] Gromadzka G., Kruszyńska M., Wierzbicka D., Litwin T., Dzieżyc K., Wierzchowska-Ciok A., Chabik G., Członkowska A. (2015). Gene Variants Encoding Proteins Involved in Antioxidant Defense System and the Clinical Expression of Wilson Disease. Liver Int..

[186] Wolfram T., Schwarz M., Reuß M., Lossow K., Ost M., Klaus S., Schwerdtle T., Kipp A.P. (2020). N-Acetylcysteine as Modulator of the Essential Trace Elements Copper and Zinc. Antioxidants.

[187] Lima J.E.B.F., Moreira N.C.S., Sakamoto-Hojo E.T. (2022). Mechanisms Underlying the Pathophysiology of Type 2 Diabetes: From Risk Factors to Oxidative Stress, Metabolic Dysfunction, and Hyperglycemia. Mutat. Res./Genet. Toxicol. Environ. Mutagen..

[188] Dludla P.V., Mabhida S.E., Ziqubu K., Nkambule B.B., Mazibuko-Mbeje S.E., Hanser S., Basson A.K., Pheiffer C., Kengne A.P. (2023). Pancreatic β-Cell Dysfunction in Type 2 Diabetes: Implications of Inflammation and Oxidative Stress. World J. Diabetes.

[189] Argaev-Frenkel L., Rosenzweig T. (2023). Redox Balance in Type 2 Diabetes: Therapeutic Potential and the Challenge of Antioxidant-Based Therapy. Antioxidants.

[190] Tiedge M., Lortz S., Drinkgern J., Lenzen S. (1997). Relation Between Antioxidant Enzyme Gene Expression and Antioxidative Defense Status of Insulin-Producing Cells. Diabetes.

[191] Grankvist K., Marklund S.L., Täljedal I.B. (1981). CuZn-Superoxide Dismutase, Mn-Superoxide Dismutase, Catalase and Glutathione Peroxidase in Pancreatic Islets and Other Tissues in the Mouse. Biochem. J..

[192] Robertson R.P. (2004). Chronic Oxidative Stress as a Central Mechanism for Glucose Toxicity in Pancreatic Islet Beta Cells in Diabetes. J. Biol. Chem..

[193] Lenzen S. (2008). Oxidative Stress: The Vulnerable β-Cell. Biochem. Soc. Trans..

[194] Bast A., Wolf G., Oberbäumer I., Walther R. (2002). Oxidative and Nitrosative Stress Induces Peroxiredoxins in Pancreatic Beta Cells. Diabetologia.

[195] Robertson R.P., Harmon J.S. (2007). Pancreatic Islet Β-cell and Oxidative Stress: The Importance of Glutathione Peroxidase. FEBS Lett..

[196] Tanaka Y., Tran P.O.T., Harmon J., Robertson R.P. (2002). A Role for Glutathione Peroxidase in Protecting Pancreatic β Cells against Oxidative Stress in a Model of Glucose Toxicity. Proc. Natl. Acad. Sci. USA.

[197] Evans J.L., Goldfine I.D., Maddux B.A., Grodsky G.M. (2002). Oxidative Stress and Stress-Activated Signaling Pathways: A Unifying Hypothesis of Type 2 Diabetes. Endocr. Rev..

[198] Hirosumi J., Tuncman G., Chang L., Görgün C.Z., Uysal K.T., Maeda K., Karin M., Hotamisligil G.S. (2023). Author Correction: A Central Role for JNK in Obesity and Insulin Resistance. Nature.

[199] Shoelson S.E., Herrero L., Naaz A. (2007). Obesity, Inflammation, and Insulin Resistance. Gastroenterology.

[200] Ma Q. (2013). Role of Nrf2 in Oxidative Stress and Toxicity. Annu. Rev. Pharmacol. Toxicol..

[201] Uruno A., Motohashi H. (2011). The Keap1–Nrf2 System as an in Vivo Sensor for Electrophiles. Nitric Oxide.

[202] Xu Z., Wei Y., Gong J., Cho H., Park J.K., Sung E.-R., Huang H., Wu L., Eberhart C., Handa J.T. (2014). NRF2 Plays a Protective Role in Diabetic Retinopathy in Mice. Diabetologia.

[203] Chartoumpekis D., Kensler T. (2013). New Player on An Old Field; the Keap1/Nrf2 Pathway as a Target for Treatment of Type 2 Diabetes and Metabolic Syndrome. CDR Curr. Diabetes Rev..

[204] Lutchmansingh F.K., Hsu J.W., Bennett F.I., Badaloo A.V., McFarlane-Anderson N., Gordon-Strachan G.M., Wright-Pascoe R.A., Jahoor F., Boyne M.S. (2018). Glutathione Metabolism in Type 2 Diabetes and Its Relationship with Microvascular Complications and Glycemia. PLoS ONE.

[205] Sekhar R.V. (2022). GlyNAC (Glycine and N-Acetylcysteine) Supplementation Improves Impaired Mitochondrial Fuel Oxidation and Lowers Insulin Resistance in Patients with Type 2 Diabetes: Results of a Pilot Study. Antioxidants.

[206] Li X., Zou J., Lin A., Chi J., Hao H., Chen H., Liu Z. (2024). Oxidative Stress, Endothelial Dysfunction, and *N* -Acetylcysteine in Type 2 Diabetes Mellitus. Antioxid. Redox Signal..

[207] Szkudlinska M.A., Von Frankenberg A.D., Utzschneider K.M. (2016). The Antioxidant N-Acetylcysteine Does Not Improve Glucose Tolerance or β-Cell Function in Type 2 Diabetes. J. Diabetes Its Complicat..

[208] Tuell D., Ford G., Los E., Stone W. (2024). The Role of Glutathione and Its Precursors in Type 2 Diabetes. Antioxidants.

[209] Dowman J.K., Tomlinson J.W., Newsome P.N. (2010). Pathogenesis of Nonalcoholic Fatty Liver Disease. QJM.

[210] Younossi Z.M., Koenig A.B., Abdelatif D., Fazel Y., Henry L., Wymer M. (2016). Global Epidemiology of Nonalcoholic Fatty Liver Disease—Meta-analytic Assessment of Prevalence, Incidence, and Outcomes. Hepatology.

[211] Li S., Tan H.-Y., Wang N., Zhang Z.-J., Lao L., Wong C.-W., Feng Y. (2015). The Role of Oxidative Stress and Antioxidants in Liver Diseases. Int. J. Mol. Sci..

[212] Seki S., Kitada T., Yamada T., Sakaguchi H., Nakatani K., Wakasa K. (2002). In Situ Detection of Lipid Peroxidation and Oxidative DNA Damage in Nonalcoholic Fatty Liver Diseases. J. Hepatol..

[213] Sanyal A.J., Campbell–Sargent C., Mirshahi F., Rizzo W.B., Contos M.J., Sterling R.K., Luketic V.A., Shiffman M.L., Clore J.N. (2001). Nonalcoholic Steatohepatitis: Association of Insulin Resistance and Mitochondrial Abnormalities. Gastroenterology.

[214] Rolo A.P., Teodoro J.S., Palmeira C.M. (2012). Role of Oxidative Stress in the Pathogenesis of Nonalcoholic Steatohepatitis. Free Radic. Biol. Med..

[215] Jaeschke H., McGill M.R., Ramachandran A. (2012). Oxidant Stress, Mitochondria, and Cell Death Mechanisms in Drug-Induced Liver Injury: Lessons Learned from Acetaminophen Hepatotoxicity. Drug Metab. Rev..

[216] Wu G., Lupton J.R., Turner N.D., Fang Y.-Z., Yang S. (2004). Glutathione Metabolism and Its Implications for Health. J. Nutr..

[217] Li L., Zhang G.-F., Lee K., Lopez R., Previs S.F., Willard B., McCullough A., Kasumov T. (2016). A Western Diet Induced NAFLD in LDLR(-/)(-) Mice Is Associated with Reduced Hepatic Glutathione Synthesis. Free Radic. Biol. Med..

[218] Marí M., Morales A., Colell A., García-Ruiz C., Fernández-Checa J.C. (2009). Mitochondrial Glutathione, a Key Survival Antioxidant. Antioxid. Redox Signal..

[219] Videla L.A., Rodrigo R., Orellana M., Fernandez V., Tapia G., Quiñones L., Varela N., Contreras J., Lazarte R., Csendes A. (2004). Oxidative Stress-Related Parameters in the Liver of Nonalcoholic Fatty Liver Disease Patients. Clin. Sci..

[220] Masarone M., Rosato V., Dallio M., Gravina A.G., Aglitti A., Loguercio C., Federico A., Persico M. (2018). Role of Oxidative Stress in Pathophysiology of Nonalcoholic Fatty Liver Disease. Oxidative Med. Cell. Longev..

[221] Santacroce G., Gentile A., Soriano S., Novelli A., Lenti M.V., Di Sabatino A. (2023). Glutathione: Pharmacological Aspects and Implications for Clinical Use in Nonalcoholic Fatty Liver Disease. Front. Med..

[222] Ucar F., Sezer S., Erdogan S., Akyol S., Armutcu F., Akyol O. (2013). The Relationship between Oxidative Stress and Nonalcoholic Fatty Liver Disease: Its Effects on the Development of Nonalcoholic Steatohepatitis. Redox Rep..

[223] Irie M., Sohda T., Anan A., Fukunaga A., Takata K., Tanaka T. (2016). Reduced Glutathione Suppresses Oxidative Stress in Nonalcoholic Fatty Liver Disease. Euroasian J. Hepato-Gastroenterol..

[224] Conde De La Rosa L., Goicoechea L., Torres S., Garcia-Ruiz C., Fernandez-Checa J.C. (2022). Role of Oxidative Stress in Liver Disorders. Livers.

[225] Arroyave-Ospina J.C., Wu Z., Geng Y., Moshage H. (2021). Role of Oxidative Stress in the Pathogenesis of Nonalcoholic Fatty Liver Disease: Implications for Prevention and Therapy. Antioxidants.

[226] Méndez-Sánchez N., Valencia-Rodríguez A., Coronel-Castillo C., Vera-Barajas A., Contreras-Carmona J., Ponciano-Rodríguez G., Zamora-Valdés D. (2020). The Cellular Pathways of Liver Fibrosis in Nonalcoholic Steatohepatitis. Ann. Transl. Med..

[227] Sutti S., Albano E. (2022). Oxidative Stress in Nonalcoholic Fatty Liver Disease: A Reappraisal of the Role in Supporting Inflammatory Mechanisms. Redox Exp. Med..

[228] Yu W., Zhang F., Meng D., Zhang X., Feng Y., Yin G., Liang P., Chen S., Liu H. (2024). Mechanism of Action and Related Natural Regulators of Nrf2 in Nonalcoholic Fatty Liver Disease. CDD Curr. Drug Deliv..

[229] Chambel S.S., Santos-Gonçalves A., Duarte T.L. (2015). The Dual Role of Nrf2 in Nonalcoholic Fatty Liver Disease: Regulation of Antioxidant Defenses and Hepatic Lipid Metabolism. BioMed Res. Int..

[230] Zhou J., Zheng Q., Chen Z. (2022). The Nrf2 Pathway in Liver Diseases. Front. Cell Dev. Biol..

[231] He Y., Jiang J., He B., Shi Z. (2021). Chemical Activators of the Nrf2 Signaling Pathway in Nonalcoholic Fatty Liver Disease. Nat. Prod. Commun..

[232] Liu J., Zheng Y., Yang S., Zhang L., Liu B., Zhang J., Yu X., Wei X., Li S., Wang J. (2024). Targeting Antioxidant Factor Nrf2 by Raffinose Ameliorates Lipid Dysmetabolism-Induced Pyroptosis, Inflammation and Fibrosis in NAFLD. Phytomedicine.

[233] Chen Z., Tian R., She Z., Cai J., Li H. (2020). Role of Oxidative Stress in the Pathogenesis of Nonalcoholic Fatty Liver Disease. Free Radic. Biol. Med..

[234] Dludla P.V., Nkambule B.B., Mazibuko-Mbeje S.E., Nyambuya T.M., Marcheggiani F., Cirilli I., Ziqubu K., Shabalala S.C., Johnson R., Louw J. (2020). N-Acetyl Cysteine Targets Hepatic Lipid Accumulation to Curb Oxidative Stress and Inflammation in NAFLD: A Comprehensive Analysis of the Literature. Antioxidants.

[235] Nguyen M.T., Lian A., Guilford F.T., Venketaraman V. (2025). A Literature Review of Glutathione Therapy in Ameliorating Hepatic Dysfunction in Nonalcoholic Fatty Liver Disease. Biomedicines.

[236] Furukawa S., Fujita T., Shimabukuro M., Iwaki M., Yamada Y., Nakajima Y., Nakayama O., Makishima M., Matsuda M., Shimomura I. (2004). Increased Oxidative Stress in Obesity and Its Impact on Metabolic Syndrome. J. Clin. Investig..

[237] Hotamisligil G.S. (2006). Inflammation and Metabolic Disorders. Nature.

[238] Gregor M.F., Hotamisligil G.S. (2011). Inflammatory Mechanisms in Obesity. Annu. Rev. Immunol..

[239] Lumeng C.N., Saltiel A.R. (2011). Inflammatory Links between Obesity and Metabolic Disease. J. Clin. Investig..

[240] Houstis N., Rosen E.D., Lander E.S. (2006). Reactive Oxygen Species Have a Causal Role in Multiple Forms of Insulin Resistance. Nature.

[241] Rains J.L., Jain S.K. (2011). Oxidative Stress, Insulin Signaling, and Diabetes. Free Radic. Biol. Med..

[242] Shoelson S.E. (2006). Inflammation and Insulin Resistance. J. Clin. Investig..

[243] Potashnik R., Bloch-Damti A., Bashan N., Rudich A. (2003). IRS1 Degradation and Increased Serine Phosphorylation Cannot Predict the Degree of Metabolic Insulin Resistance Induced by Oxidative Stress. Diabetologia.

[244] Acosta-Martinez M., Cabail M.Z. (2022). The PI3K/Akt Pathway in Meta-Inflammation. Int. J. Mol. Sci..

[245] Chen C.-A., De Pascali F., Basye A., Hemann C., Zweier J.L. (2013). Redox Modulation of Endothelial Nitric Oxide Synthase by Glutaredoxin-1 through Reversible Oxidative Post-Translational Modification. Biochemistry.

[246] Panieri E., Santoro M.M. (2015). ROS Signaling and Redox Biology in Endothelial Cells. Cell. Mol. Life Sci..

[247] Zalba G., José G.S., Moreno M.U., Fortuño M.A., Fortuño A., Beaumont F.J., Díez J. (2001). Oxidative Stress in Arterial Hypertension: Role of NAD(P)H Oxidase. Hypertension.

[248] Marasinghe C.K., Je J. (2025). Glutathione as a Therapeutic Agent for OxLDL-Induced Endothelial Dysfunction and Atherosclerosis Prevention. Cell Biol. Int..

[249] Tousoulis D., Kampoli A.-M., Tentolouris Nikolaos Papageorgiou C., Stefanadis C. (2012). The Role of Nitric Oxide on Endothelial Function. CVP Curr. Vasc. Pharmacol..

[250] Jiang H., Zhou Y., Nabavi S.M., Sahebkar A., Little P.J., Xu S., Weng J., Ge J. (2022). Mechanisms of Oxidized LDL-Mediated Endothelial Dysfunction and Its Consequences for the Development of Atherosclerosis. Front. Cardiovasc. Med..

[251] Hansson G.K. (2005). Inflammation, Atherosclerosis, and Coronary Artery Disease. N. Engl. J. Med..

[252] Madamanchi N.R., Vendrov A., Runge M.S. (2005). Oxidative Stress and Vascular Disease. ATVB Arterioscler. Thromb. Vasc. Biol..

[253] Di Pietro N., Formoso G., Pandolfi A. (2016). Physiology and Pathophysiology of oxLDL Uptake by Vascular Wall Cells in Atherosclerosis. Vasc. Pharmacol..

[254] Yang X., Li Y., Li Y., Ren X., Zhang X., Hu D., Gao Y., Xing Y., Shang H. (2017). Oxidative Stress-Mediated Atherosclerosis: Mechanisms and Therapies. Front. Physiol..

[255] Aviram M. (1996). Interaction of Oxidized Low Density Lipoprotein with Macrophages in Atherosclerosis, and the Antiatherogenicity of Antioxidants. Eur. J. Clin. Chem. Clin. Biochem..

[256] Valko M., Leibfritz D., Moncol J., Cronin M.T.D., Mazur M., Telser J. (2007). Free Radicals and Antioxidants in Normal Physiological Functions and Human Disease. Int. J. Biochem. Cell Biol..

[257] Westhuyzen J. (1997). The Oxidation Hypothesis of Atherosclerosis: An Update. Ann. Clin. Lab. Sci..

[258] Zhang Z., Zhou S., Jiang X., Wang Y.-H., Li F., Wang Y.-G., Zheng Y., Cai L. (2015). The Role of the Nrf2/Keap1 Pathway in Obesity and Metabolic Syndrome. Rev. Endocr. Metab. Disord..

[259] Costa V.B., De Matos I.A.F., Nogueira I.R.G., De Godoi M.A., Leite F.R.M., Guimarães-Stabili M.R. (2026). Nrf2 Activation in Inflammatory Diseases: A Review of Natural and Synthetic Modulators. Oxidative Med. Cell. Longev..

[260] Da Costa R.M., Rodrigues D., Pereira C.A., Silva J.F., Alves J.V., Lobato N.S., Tostes R.C. (2019). Nrf2 as a Potential Mediator of Cardiovascular Risk in Metabolic Diseases. Front. Pharmacol..

[261] Ungvari Z., Kaley G., De Cabo R., Sonntag W.E., Csiszar A. (2010). Mechanisms of Vascular Aging: New Perspectives. J. Gerontol. Ser. A Biol. Sci. Med. Sci..

[262] Hou H., Qin X., Li G., Cui Z., Zhang J., Dong B., Wang Z., Zhao H. (2024). Nrf2-Mediated Redox Balance Alleviates LPS-Induced Vascular Endothelial Cell Inflammation by Inhibiting Endothelial Cell Ferroptosis. Sci. Rep..

[263] Robertson R.P. (2023). Nrf2 and Antioxidant Response in Animal Models of Type 2 Diabetes. Int. J. Mol. Sci..

[264] Ungvari Z., Bagi Z., Feher A., Recchia F.A., Sonntag W.E., Pearson K., De Cabo R., Csiszar A. (2010). Resveratrol Confers Endothelial Protection via Activation of the Antioxidant Transcription Factor Nrf2. Am. J. Physiol. -Heart Circ. Physiol..

[265] Matuz-Mares D., Riveros-Rosas H., Vilchis-Landeros M.M., Vázquez-Meza H. (2021). Glutathione Participation in the Prevention of Cardiovascular Diseases. Antioxidants.

[266] Tan M., Yin Y., Ma X., Zhang J., Pan W., Tan M., Zhao Y., Yang T., Jiang T., Li H. (2023). Glutathione System Enhancement for Cardiac Protection: Pharmacological Options against Oxidative Stress and Ferroptosis. Cell Death Dis..

[267] Sahasrabudhe S.A., Terluk M.R., Kartha R.V. (2023). N-Acetylcysteine Pharmacology and Applications in Rare Diseases—Repurposing an Old Antioxidant. Antioxidants.

[268] Rushworth G.F., Megson I.L. (2014). Existing and Potential Therapeutic Uses for N-Acetylcysteine: The Need for Conversion to Intracellular Glutathione for Antioxidant Benefits. Pharmacol. Ther..

[269] Hashimoto S., Gon Y., Matsumoto K., Takeshita I., Horie T. (2001). N-acetylcysteine Attenuates TNF-α-induced P38 MAP Kinase Activation and P38 MAP Kinase-mediated IL-8 Production by Human Pulmonary Vascular Endothelial Cells. Br. J. Pharmacol..

[270] Santus P., Signorello J.C., Danzo F., Lazzaroni G., Saad M., Radovanovic D. (2024). Anti-Inflammatory and Anti-Oxidant Properties of N-Acetylcysteine: A Fresh Perspective. JCM J. Clin. Med..

[271] Faghfouri A.H., Zarezadeh M., Tavakoli-Rouzbehani O.M., Radkhah N., Faghfuri E., Kord-Varkaneh H., Tan S.C., Ostadrahimi A. (2020). The Effects of N-Acetylcysteine on Inflammatory and Oxidative Stress Biomarkers: A Systematic Review and Meta-Analysis of Controlled Clinical Trials. Eur. J. Pharmacol..

[272] Khalatbari Mohseni G., Hosseini S.A., Majdinasab N., Cheraghian B. (2023). Effects of N-acetylcysteine on Oxidative Stress Biomarkers, Depression, and Anxiety Symptoms in Patients with Multiple Sclerosis. Neuropsychopharmacol. Rep..

[273] Rani M., Aggarwal R., Vohra K. (2020). Effect of N-Acetylcysteine on Metabolic Profile in Metabolic Syndrome Patients. Metab. Syndr. Relat. Disord..

[274] McCarty M.F., O’Keefe J.H., DiNicolantonio J.J. (2018). Dietary Glycine Is Rate-Limiting for Glutathione Synthesis and May Have Broad Potential for Health Protection. Ochsner J..

[275] Taniguchi M., Harm T. (1983). Effects of Riboflavin and Selenium Deficiencies on Glutathione and Its Relating Enzyme Activities with Respect to Lipid Peroxide Content of Rat Livers. J. Nutr. Sci. Vitaminol..

[276] Houghton C.A., Fassett R.G., Coombes J.S. (2016). Sulforaphane and Other Nutrigenomic Nrf2 Activators: Can the Clinician’s Expectation Be Matched by the Reality?. Oxidative Med. Cell. Longev..

[277] Kim A.D., Zhang R., Kang K.A., You H.J., Hyun J.W. (2011). Increased Glutathione Synthesis Following Nrf2 Activation by Vanadyl Sulfate in Human Chang Liver Cells. Int. J. Mol. Sci..

[278] Dai J., Jones D.P., Goldberg J., Ziegler T.R., Bostick R.M., Wilson P.W., Manatunga A.K., Shallenberger L., Jones L., Vaccarino V. (2008). Association between Adherence to the Mediterranean Diet and Oxidative Stress. Am. J. Clin. Nutr..

[279] Bellanti F., Lo Buglio A., Dobrakowski M., Kasperczyk A., Kasperczyk S., Serviddio G., Vendemiale G. (2024). Adherence to Mediterranean Diet and Biomarkers of Redox Balance and Inflammation in Old Patients Hospitalized in Internal Medicine. Nutrients.

[280] Mardinoglu A., Shoaie S., Bergentall M., Ghaffari P., Zhang C., Larsson E., Bäckhed F., Nielsen J. (2015). The Gut Microbiota Modulates Host Amino Acid and Glutathione Metabolism in Mice. Mol. Syst. Biol..

[281] Wu X., Mu B., Li G., Du R., Park S. (2026). Gut Microbial Composition, Oxidative Stress, and Immunity in Metabolic Disease: Toward Personalized Interventions. Antioxidants.

[282] Schultz M., Dutta S., Tew K.D. (1997). Inhibitors of Glutathione S-Transferases as Therapeutic Agents. Adv. Drug Deliv. Rev..

[283] Cai P.-Y., Ma M.-L., Zhang Y.-F., Zhou Z.-X., Wang Y., He L.-P., Wang W. (2022). Inhibition of Glutathione Metabolism Can Limit the Development of Pancreatic Cancer. WJSC World J. Stem Cells.

[284] Steele M.L., Fuller S., Patel M., Kersaitis C., Ooi L., Münch G. (2013). Effect of Nrf2 Activators on Release of Glutathione, Cysteinylglycine and Homocysteine by Human U373 Astroglial Cells. Redox Biol..

[285] Bellezza I., Giambanco I., Minelli A., Donato R. (2018). Nrf2-Keap1 Signaling in Oxidative and Reductive Stress. Biochim. Biophys. Acta (BBA)—Mol. Cell Res..

[286] Crisman E., Duarte P., Dauden E., Cuadrado A., Rodríguez-Franco M.I., López M.G., León R. (2023). KEAP1-NRF2 Protein–Protein Interaction Inhibitors: Design, Pharmacological Properties and Therapeutic Potential. Med. Res. Rev..

[287] Jiang X., Zheng H., Zhang X., Tang M., Peng J., Cai X., Su K., Zhang R., Ye N., Lin L. (2026). Covalent Modification of Keap1 Cys489 by NU6300 Activates Nrf2 Signaling and Suppresses NLRP3 Inflammasome-Mediated Pyroptosis. J. Pharm. Anal..

[288] Brennan M.S., Matos M.F., Li B., Hronowski X., Gao B., Juhasz P., Rhodes K.J., Scannevin R.H. (2015). Dimethyl Fumarate and Monoethyl Fumarate Exhibit Differential Effects on KEAP1, NRF2 Activation, and Glutathione Depletion In Vitro. PLoS ONE.

[289] Loewe R., Holnthoner W., Gröger M., Pillinger M., Gruber F., Mechtcheriakova D., Hofer E., Wolff K., Petzelbauer P. (2002). Dimethylfumarate Inhibits TNF-Induced Nuclear Entry of NF-κB/P65 in Human Endothelial Cells. J. Immunol..

[290] Linker R.A., Lee D.-H., Ryan S., Van Dam A.M., Conrad R., Bista P., Zeng W., Hronowsky X., Buko A., Chollate S. (2011). Fumaric Acid Esters Exert Neuroprotective Effects in Neuroinflammation via Activation of the Nrf2 Antioxidant Pathway. Brain.

[291] Jain S.K., Micinski D. (2013). Vitamin D Upregulates Glutamate Cysteine Ligase and Glutathione Reductase, and GSH Formation, and Decreases ROS and MCP-1 and IL-8 Secretion in High-Glucose Exposed U937 Monocytes. Biochem. Biophys. Res. Commun..

[292] McElroy P.B., Sri Hari A., Day B.J., Patel M. (2017). Post-Translational Activation of Glutamate Cysteine Ligase with Dimercaprol. J. Biol. Chem..

[293] Trivedi M.S., Holger D., Bui A.T., Craddock T.J.A., Tartar J.L. (2017). Short-Term Sleep Deprivation Leads to Decreased Systemic Redox Metabolites and Altered Epigenetic Status. PLoS ONE.

[294] Jiao Y., Wang Y., Guo S., Wang G. (2017). Glutathione Peroxidases as Oncotargets. Oncotarget.

[295] Singh R.R., Reindl K.M. (2021). Glutathione S-Transferases in Cancer. Antioxidants.

[296] Corti A., Belcastro E., Dominici S., Maellaro E., Pompella A. (2020). The Dark Side of Gamma-Glutamyltransferase (GGT): Pathogenic Effects of an ‘Antioxidant’ Enzyme. Free Radic. Biol. Med..

[297] Wilkins H.M., Kirchhof D., Manning E., Joseph J.W., Linseman D.A. (2013). Mitochondrial Glutathione Transport Is a Key Determinant of Neuronal Susceptibility to Oxidative and Nitrosative Stress. J. Biol. Chem..

[298] Cacciatore I., Cornacchia C., Pinnen F., Mollica A., Di Stefano A. (2010). Prodrug Approach for Increasing Cellular Glutathione Levels. Molecules.

[299] Yin N., Harris P.W.R., Liu M., Sun J., Chen G., Wen J., Brimble M.A. (2025). Enhancing the Oral Bioavailability of Glutathione Using Innovative Analogue Approaches. Pharmaceutics.

[300] Li T., Wang H., Zhang H., Zhang P., Zhang M., Feng H., Duan X., Liu W., Wang X., Sun Z. (2023). Effect of Breathing Exercises on Oxidative Stress Biomarkers in Humans: A Systematic Review and Meta-Analysis. Front. Med..

[301] Budkowska M., Cecerska-Heryć E., Marcinowska Z., Siennicka A., Dołęgowska B. (2022). The Influence of Circadian Rhythm on the Activity of Oxidative Stress Enzymes. Int. J. Mol. Sci..

[302] Davinelli S., Medoro A., Savino R., Scapagnini G. (2024). Sleep and Oxidative Stress: Current Perspectives on the Role of NRF2. Cell Mol. Neurobiol..

[303] Pļaviņa L., Edelmers E. (2025). Oxidative Stress Modulation and Glutathione System Response During a 10-Day Multi-Stressor Field Training. JFMK J. Funct. Morphol. Kinesiol..

[304] Radak Z., Chung H.Y., Koltai E., Taylor A.W., Goto S. (2008). Exercise, Oxidative Stress and Hormesis. Ageing Res. Rev..

[305] Vargas-Mendoza N., Morales-González Á., Madrigal-Santillán E.O., Madrigal-Bujaidar E., Álvarez-González I., García-Melo L.F., Anguiano-Robledo L., Fregoso-Aguilar T., Morales-Gonzalez J.A. (2019). Antioxidant and Adaptative Response Mediated by Nrf2 during Physical Exercise. Antioxidants.

[306] Merry T.L., Ristow M. (2016). Mitohormesis in Exercise Training. Free Radic. Biol. Med..

[307] Villafuerte G., Miguel-Puga A., Murillo Rodríguez E., Machado S., Manjarrez E., Arias-Carrión O. (2015). Sleep Deprivation and Oxidative Stress in Animal Models: A Systematic Review. Oxidative Med. Cell. Longev..

[308] Von Bohlen Und Halbach O. (2022). Controlling Glutathione Entry into Mitochondria: Potential Roles for SLC25A39 in Health and (Treatment of) Disease. Signal Transduct. Target. Ther..

[309] Devóz P.P., Reis M.B.D., Gomes W.R., Maraslis F.T., Ribeiro D.L., Antunes L.M.G., Batista B.L., Grotto D., Reis R.M., Barbosa F. (2021). Adaptive Epigenetic Response of Glutathione (GSH)-Related Genes against Lead (Pb)-Induced Toxicity, in Individuals Chronically Exposed to the Metal. Chemosphere.

[310] Deng J., Li Y., Yin L., Liu S., Li Y., Liao W., Mu L., Luo X., Qin J. (2025). Histone Lactylation Enhances GCLC Expression and Thus Promotes Chemoresistance of Colorectal Cancer Stem Cells through Inhibiting Ferroptosis. Cell Death Dis..

[311] Huang X., Su Z., Li J., He J., Zhao N., Nie L., Guan B., Huang Q., Zhao H., Lu G.-D. (2023). Downregulation of LncRNA GCLC-1 Promotes Microcystin-LR-Induced Malignant Transformation of Human Liver Cells by Regulating GCLC Expression. Toxics.

[312] Zhu X., Chen Z., Shen W., Huang G., Sedivy J.M., Wang H., Ju Z. (2021). Inflammation, Epigenetics, and Metabolism Converge to Cell Senescence and Ageing: The Regulation and Intervention. Signal Transduct. Target. Ther..

